# Monitoring G protein-coupled receptor and β-arrestin trafficking in live cells using enhanced bystander BRET

**DOI:** 10.1038/ncomms12178

**Published:** 2016-07-11

**Authors:** Yoon Namkung, Christian Le Gouill, Viktoria Lukashova, Hiroyuki Kobayashi, Mireille Hogue, Etienne Khoury, Mideum Song, Michel Bouvier, Stéphane A. Laporte

**Affiliations:** 1 Department of Medicine, Research Institute of the McGill University Health Center (RI-MUHC), McGill University, Montréal, Québec, Canada H4A 3J1; 2 Department of Biochemistry and Institute for Research in Immunology and Cancer (IRIC), Université de Montréal, Montréal, Québec, Canada H3C 1J4; 3 Department of Pharmacology and Therapeutics, McGill University, Montréal, Québec, Canada H3G 1Y6; 4 Department of Anatomy and Cell Biology, McGill University, Montréal, Québec, Canada H3A 0C7

## Abstract

Endocytosis and intracellular trafficking of receptors are pivotal to maintain physiological functions and drug action; however, robust quantitative approaches are lacking to study such processes in live cells. Here we present new bioluminescence resonance energy transfer (BRET) sensors to quantitatively monitor G protein-coupled receptors (GPCRs) and β-arrestin trafficking. These sensors are based on bystander BRET and use the naturally interacting chromophores luciferase (RLuc) and green fluorescent protein (rGFP) from *Renilla*. The versatility and robustness of this approach are exemplified by anchoring rGFP at the plasma membrane or in endosomes to generate high dynamic spectrometric BRET signals on ligand-promoted recruitment or sequestration of RLuc-tagged proteins to, or from, specific cell compartments, as well as sensitive subcellular BRET imaging for protein translocation visualization. These sensors are scalable to high-throughput formats and allow quantitative pharmacological studies of GPCR trafficking in real time, in live cells, revealing ligand-dependent biased trafficking of receptor/β-arrestin complexes.

## Abstract (web summary)

Cellular signaling processes often involve trafficking of receptors and other proteins between subcellular compartments. Here the authors demonstrate a method based on the concept of Enhanced bystander Bioluminescence Resonance Energy Transfer (EbBRET) that allows efficient real time monitoring of endocytosis and trafficking.

## Graphical abstract

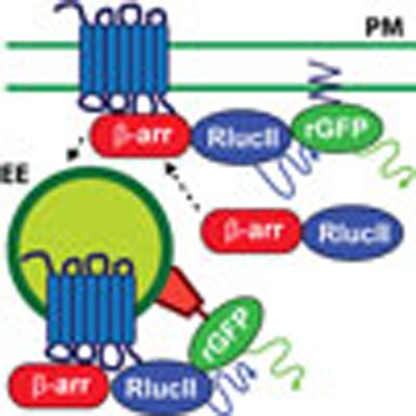

Trafficking of receptors to, or from the plasma membrane (PM), as well as their targeting to specialized intracellular compartments are pivotal processes to maintain cellular integrity. Signalling functions of G protein-coupled receptors (GPCRs) are tightly regulated by endocytosis, the targeting of receptors to endosomes and their sorting to lysosomes or recycling to the PM^1^,^2^. Potential therapeutic benefits can be obtained for drugs promoting intracellular signalling; while for other receptors, their recycling and/or proper trafficking to the PM is essential to maintain physiological functions. Recent findings suggest that receptor trafficking can be differentially regulated by ligands^3^,^4^, potentially affecting cell responsiveness and drug efficacies. Moreover, for naturally occurring mutations in GPCRs that poorly traffic to the PM, the use of pharmacological chaperones (PCs) can rescue receptor functions at the PM and ensuing cellular responses^5^,^6^,^7^. Advantages can thus be obtained, both in drug and basic research discovery programs, from developing means to efficiently study receptor trafficking. Classical approaches, however, often lack sensitivity, are semi-quantitative, and are not always amenable for a short time scales (that is, within seconds to minutes), or long continued observations (tens of minutes to hours) and/or for quantitative high-throughput monitoring of receptor trafficking in living cells. Image-based fluorescence microscopy—despite offering temporal resolution and being scalable in high throughput—requires sophisticated algorithms for quantitative analysis of receptor trafficking.

Bioluminescence resonance energy transfer (BRET) approaches, which are quantitative, have been extensively used to study protein–protein interactions^8^,^9^,^10^ and receptor signalling^9^,^11^. BRET relies on the transfer of energy between a bioluminescent donor, such as luciferase, and a fluorescent acceptor like green fluorescent protein (GFP). Conventional BRET assays use the non-natural combination of luciferase from *Renilla reniformis* (Rluc) and GFP variants from *Aequorea victoria*, which do not interact spontaneously, hence, limiting non-specific signals that originate from random interactions (that is, also referred to as bystander BRET). Bystander BRET using non-natural chromophores also present possibilities to monitor the proximity of proteins in the same subcellular compartment, and has been successfully used to vet receptor trafficking^3^,^12^,^13^,^14^,^15^,^16^. In this case, the specificity of the response is imparted by the re-localization of either the Rluc- or GPF-tagged proteins in cells, which modulates BRET signals.

On the other hand, the natural combination of Rluc and GFP from *Renilla reniformis* (rGFP), which self-associate with moderate affinity and optimally transfer energy^10^,^17^,^18^, have been used as BRET pair to improve signal^10^,^19^. Here we took advantage of the characteristics of these naturally interacting chromophores from *Renilla reniformis* to develop new, highly dynamic BRET-trafficking sensors (herein after referred to as ‘enhanced bystander BRET' or EbBRET). We show that these sensors improve BRET signals for quantitative monitoring of changes in concentration of donor-tagged proteins relative to the acceptors anchored in either the same or distinct cellular compartments in live cells, allowing to dynamically study, with high sensitivity, GPCR internalization and intracellular trafficking of receptors and β-arrestin (including translocation to and from the PM, targeting to different endosomes and receptor recycling to the PM), as well as forward GPCR trafficking. The sensitivity and selectivity of the sensors improve both spectrometric and microscopy-based studies of ligand promoted and biased trafficking, as well as high-throughput screening (HTS).

## Results

### Characterization of new EbBRET sensors

**Figure 1:**
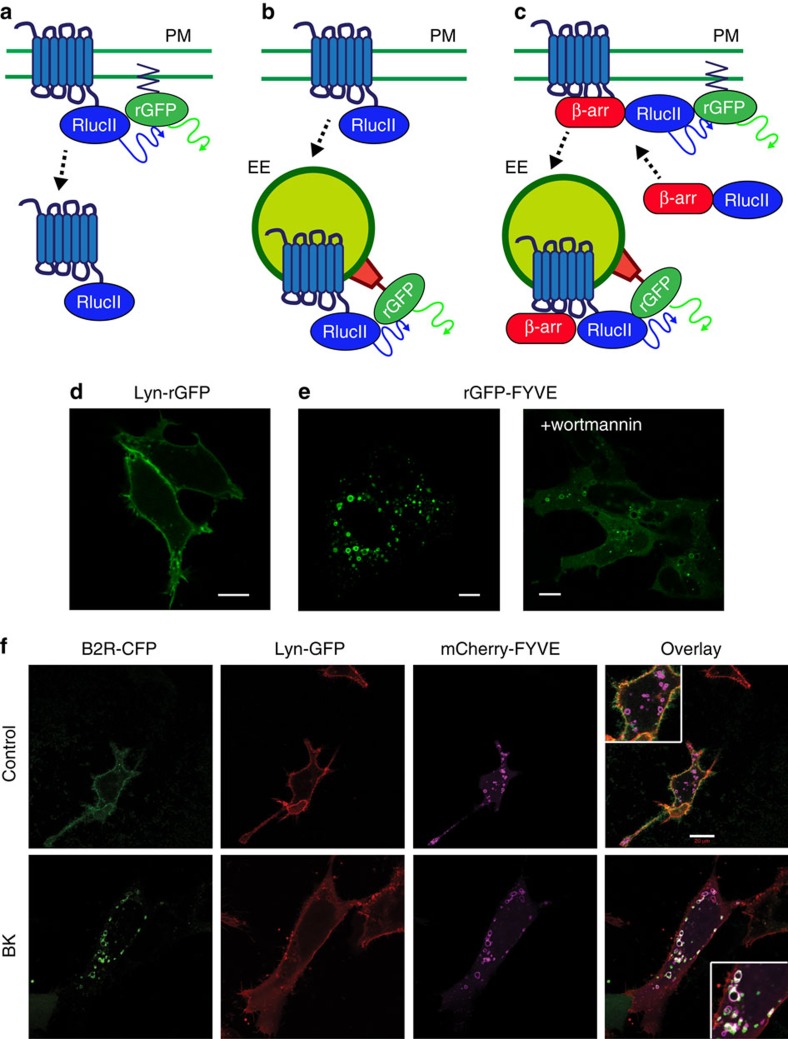
Characterization of EbBRET-based trafficking sensors. (**a**–**c**) Illustration of three BRET-based sensor configurations for monitoring GPCR and β-arrestin trafficking. (**a**) To monitor the receptor amount at the plasma membrane (PM), rGFP was anchored at the PM by tagging the acylation motif of Lyn-kinase (MGCIKSKGKDS) to the N terminus of rGFP. (**b**,**c**) To examine the targeting of receptor (**b**) or β-arrestin (**c**) to the early endosome (EE), the FYVE domain of endofin (Q739-K806), which tethers the sensor in the EE, was fused to the C terminus of rGFP. The PM- and EE-targeted rGFP are the EbBRET acceptor for RlucII-tagged GPCRs and β-arrestin donors. (**d**,**e**) Confocal fluorescence microscopy of HEK293 cells expressing either Lyn-rGFP (**d**) or rGFP-FYVE (**e**). rGFP-FYVE expressing cells were treated with 500 nM wortmannin for 40 min (**e**, right panel). (**f**) Validation of the subcellular localization of the PM-targeted Lyn and EE-targeted FYVE domains during receptor endocytosis. Confocal fluorescence microscopy of HEK293 cells transiently expressing B2R-CFP, Lyn-GFP10 and mCherry-FYVE. Top panels: basal condition (control); bottom panels: following a 15-min stimulation with 1 μM bradykinin (BK). For enhanced co-localization visualization, pseudo colours for B2R-GFP (green), Lyn-GFP10 (red) and mCherry-FYVE (purple) were applied. Scale bars, 10 μm (d,e) and 20 μm (f).

We engineered new BRET acceptors expressed at either the PM or in early endosomes (EEs) using rGFP, partnered with a highly luminescent mutant form of Rluc (RlucII, also known as Rluc3)^20^, as the donor tagged to either GPCRs or β-arrestins (Fig. 1a–c). rGFP was either fused through its N terminus to the fatty acylation motif of Lyn-kinase or through its C terminus to the polybasic sequence and prenylation CAAX box of KRas (Lyn-rGFP or rGFP-CAAX) for their targeting to the PM^21^. For targeting to EEs, the FYVE domain of endofin, which binds phosphatidylinositol 3-phosphate (PI3P) in EEs^22^, was attached at the C terminus of rGFP (rGFP-FYVE). As expected, Lyn-rGFP and rGFP-CAAX are localized at the PM when expressed in HEK293 cells (Fig. 1d; Supplementary Fig. 1a), whereas rGFP-FYVE is selectively localized in intracellular vesicles (Fig. 1e, left panel) where it co-localized with Rab5, a marker of EEs (Supplementary Fig. 1b). Blocking PI3P generation using the PI3K inhibitors wortmannin or LY294002, de-localized the endosomal BRET acceptor to the cytosol (right panels, Fig. 1e; Supplementary Fig. 1c, respectively) confirming the specificity of the PI3P-binding domain of rGFP-FYVE to EEs. To visualize the behaviours of the sensors in the trafficking of GPCRs, we co-expressed the bradykinin (BK) B2 receptor (B2R), a GPCR that traffics to EEs^23^, which was tagged at its C terminus with cyan fluorescent protein (B2R-CFP), with both Lyn-GFP and a mCherry-tagged version of the FYVE domain (mCherry-FYVE; Fig. 1f). Under basal conditions, B2R and Lyn-GFP signals co-localized at the PM (top panels). On agonist stimulation, the B2R signal separated from Lyn-GFP, and receptors segregated into EEs where they co-localized with mCherry-FYVE (bottom panels). B2R moved from one cellular compartment to another on agonist stimulation, while both the PM and the endosomal makers (that is, Lyn-GFP and mCherry-FYVE, respectively) largely remained in their respective cellular compartments. Similar results were obtained for two other GPCRs, the angiotensin II type 1 receptor (AT1R) and the β2-adreneric receptor (β2AR) that also internalize and traffic to EEs (Supplementary Fig. 2)^4^,^24^,^25^.

### Using RLuc:rGFP BRET pair to monitor protein trafficking

**Figure 2:**
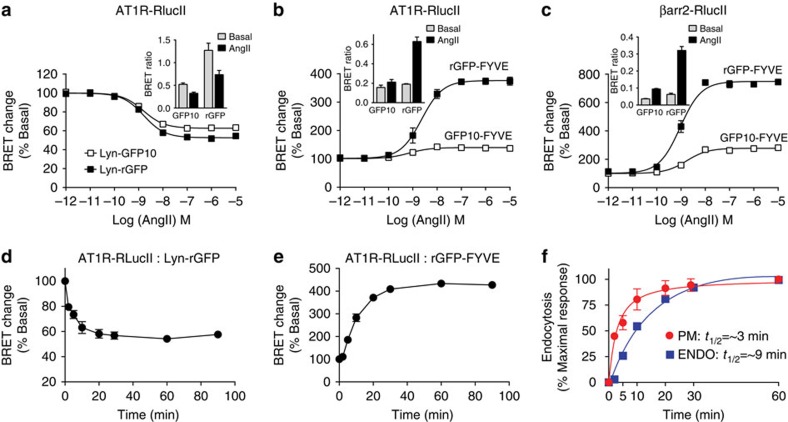
Agonist- and time-dependent trafficking of AT1R and β-arrestin2 measured by EbBRET. HEK293 cells were transfected with the indicated EbBRET pairs. To monitor AT1R-mediated βarr2-RlucII trafficking, 3 μg of AT1R was co-transfected in addition to the indicated BRET pairs in **c**. Cells were incubated with the indicated concentrations of AngII for 30 min (**a**), or 40 min (**b**,**c**), and BRET signals were measured as described in the Methods section. The BRET ratio changes on agonist treatment are expressed as a percentage of BRET ratio observed in the control (without AngII) group. (**a**–**c**, inset) Raw BRET ratios in the absence (basal) or presence of 100 nM AngII were plotted as bar graphs. Data are represented as the means±s.e.m. of three independent experiments. (**d**,**e**) Cells were incubated in the absence or presence of 100 nM AngII at 37 °C for the indicated times before BRET measurements. The BRET ratio changes on agonist treatment are expressed as a percentage of BRET ratio observed in the control (without AngII) group. Data are represented as the means±s.e.m. of three independent experiments. (**f**) Data in **d** and **e** were normalized to their respective maximal responses and plotted in the same graph. The *t*_1/2_ for maximal responses were calculated using a single-exponential fit using GraphPad Prism.

Given the proper distribution and behaviour of the rGFP- and RLuc-based biosensors, we next assessed their use for quantifying trafficking of receptors and β-arrestin. The advantage of the Rluc:rGFP BRET pair over the conventional BRET1 (Rluc:venus/YFP) and BRET2 (Rluc:GFP2/GFP10) is illustrated in Supplementary Fig. 3. Fusion between the RlucII and the different GFP acceptors reveals a much greater efficiency of energy transfer to the rGFP acceptor than with the non-natural variants (Supplementary Fig. 3b). To assess the sensitivity of Rluc:rGFP BRET biosensors, the AT1R fused to RlucII at its C terminus (AT1R-RlucII) was used as a prototypical receptor undergoing β-arrestin-dependent internalization and EEs co-trafficking^4^,^24^. Similar to the untagged receptor, AT1R-RlucII proficiently internalized, as illustrated in Supplementary Fig. 4 using a classical radioligand-binding assay. Agonist-mediated sequestration of AT1R from the PM was not altered by co-expressing Lyn-rGFP (Supplementary Fig. 4a). Internalization of AT1R-RlucII was increased by overexpressing β-arrestin2, and inhibited with the dominant negative K44A mutant form of dynamin (DynK44A; Supplementary Fig. 4b), consistent with β-arrestins' role in agonist-dependent, clathrin-mediated internalization of AT1R^26^,^27^,^28^. Expression of AT1R-RlucII and Lyn-rGFP produced a high basal EbBRET signal, in agreement with their co-localization and enrichment at the PM, which favours bystander encounter between the donor and acceptor (Fig. 2a). Angiotensin II (AngII)-mediated sequestration of receptors, which decreased the donor:acceptor ratio at the PM, reduced the EbBRET signal in an agonist-dependent manner (Log of effector concentration for half-maximum responses (logEC_50_) of −8.76±0.31). Similar responses were observed using another rGFP-tagged PM marker (rGFP-CAAX) with AT1R-RlucII (logEC_50_ of −9.16±0.35; Supplementary Fig. 5a, with potencies not significantly different from that obtained using Lyn-rGFP (*P* value=0.4356)). Concentration-dependent decrease in BRET signals between other RlucII-tagged GPCRs, such as B2R and β2AR, and Lyn-rGFP were also observed upon agonist-mediated stimulation of receptors (Supplementary Fig. 5b), although with different amplitudes.

Since β-arrestin is required for the initial steps of endocytosis^28^, we also validated that βarr2-RlucII translocated to the PM upon AT1R activation by EbBRET, using either Lyn-rGFP or rGFP-CAAX. AngII-dependent increases in BRET ratios were observed with both acceptor sensors. rGFP-CAAX generated higher EbBRET ratios than Lyn-rGFP, but produced similar EC_50_s (Supplementary Fig. 5c; Lyn-rGFP: logEC_50_ of −8.28±0.08 and rGFP-CAAX: logEC_50_ of −8.22±0.09), confirming that both PM markers can be used to monitor the translocation of β-arrestin and the sequestration of receptors. Similar responses were also obtained with the β2AR (Supplementary Fig. 5d), another GPCR that recruits β-arrestin for its internalization^28^, but does not co-internalize with it in EEs^24^. However, prostaglandin F2α-mediated stimulation of prostaglandin F receptor, which is known not to promote β-arrestin binding nor to efficiently internalize^29^, did not induce any BRET signal increase (Supplementary Fig. 5e). The rGFP-CAAX sensor was also sensitive enough to detect β-arrestin2 translocation to the PM following endogenous receptor activation (Supplementary Fig. 5f). Indeed, stimulation of HEK293 cells, which endogenously expressed the GPCR protease-activated receptor 2 (PAR2)^30^, with the PAR2 selective agonist SLIKGV, dose dependently increased the BRET signal between βarr2-RlucII and rGFP-CAAX (Supplementary Fig. 5f). The BRET signal between β-arrestin2 and rGFP-CAAX could also be observed upon stimulation of β2AR-expressing HEK293 cells with the weak partial agonist, salbutamol (Supplementary Fig. 5g). β-arrestin2 translocation to the PM on activation of the endogenously expressed PAR2 or partial β2AR agonist was not detectable using the βarr2-RlucII:GFP10-CAAX pair, illustrating the higher sensitivity of the RlucII:rGFP-based (EbBRET) sensor over the RlucII:GFP10 (BRET2) pair.

We next assessed the trafficking of receptors and β-arrestin to EEs using EbBRET sensors. Consistent with the agonist-mediated trafficking of AT1R and β-arrestin2 to EEs^4^,^24^ and ensuing increase of the donor concentration in that compartment, we observed an increase in the BRET signal in cells expressing either AT1R-RlucII, or AT1R and βarr2-RlucII, with rGFP-FYVE; with potencies similar to that of β-arrestin recruitment to the PM and of receptor internalization (Fig. 2b,c; logEC_50_ of −8.62±0.14 and −9.05±0.11, respectively). Importantly, expressing rGFP with either RlucII-tagged receptor or βarr2-RlucII generated larger absolute bystander BRET signals in all assays, when compared with the RlucII:GFP10 BRET pairs (Fig. 2a–c; Supplementary Fig. 5a,c,d,f,g). When expressed as % of the basal BRET signal to take into account differences in basal signal amplitude, normalized BRET changes were comparable between GFP10 and rGFP when assessing receptor removal from the PM (Fig. 2a; Supplementary Fig. 5a; although the rGFP consistently performed better than the GFP10), but were much greater with rGFP when monitoring agonist-promoted targeting of receptor, and β-arrestin to EEs and β-arrestin translocation to the PM (Fig. 2b,c; Supplementary Fig. 5c,d), hence, revealing a greater dynamic window. Agonist-promoted increase in BRET signals between receptor-RlucII and rGFP-FYVE were also observed for β2AR and B2R (Supplementary Fig. 6a). For each receptor, EC_50_s of the agonist-promoted loss of PM receptor and their appearance in EEs were almost identical (for example, loss of PM receptors: logEC_50_ for AngII-AT1R: −8.97±0.17; BK-B2R: −8.55±0.13; and isoproterenol (Iso)-β2AR: −7.61±0.20; accumulation in EE: AngII-AT1R: −9.12±0.10; BK-B2R: −9.00±0.11 and Iso-β2AR: −7.61±0.10, respectively), confirming the quantitative robustness of the assays. Stimulation of B2R, which like AT1R traffics to EEs with β-arrestin^23^, also resulted in a robust concentration-dependent increase of the BRET signal between βarr2-RlucII and rGFP-FYVE (Supplementary Fig. 6b). In contrast, agonist-mediated stimulation of β2AR or prostaglandin F receptor did not promote BRET signals between βarr2-RlucII and rGFP-FYVE (Supplementary Fig. 6b), consistent with the known behaviour of these receptors^24^,^29^.

**Figure 3:**
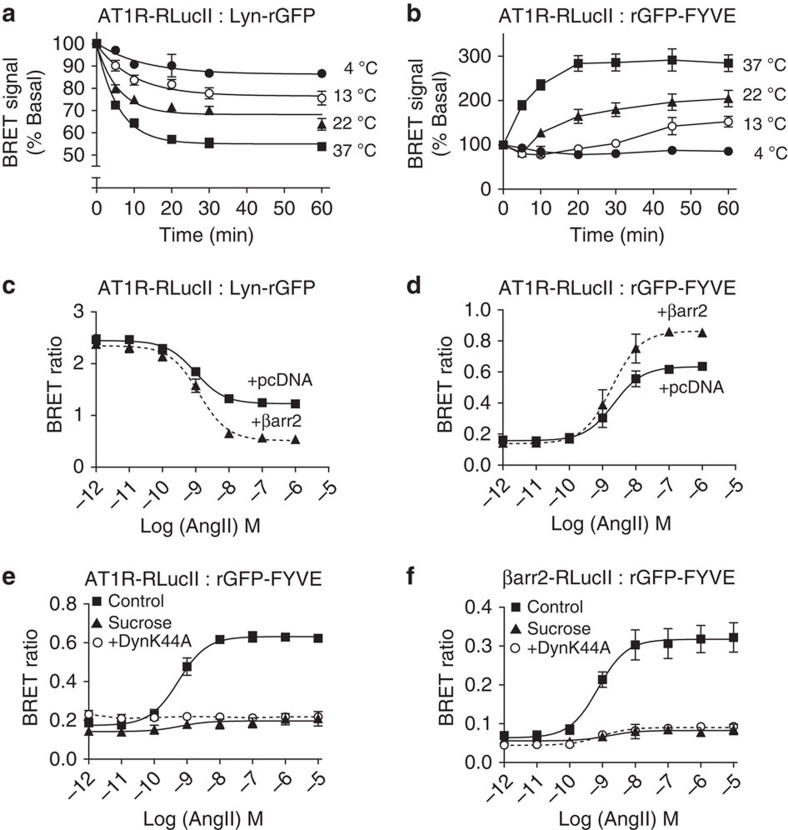
AngII-mediated AT1R and β-arrestin2 trafficking assessed by EbBRET. (**a**,**b**) HEK293 cells were transfected with AT1R-RlucII along with either Lyn-rGFP (**a**) or rGFP-FYVE (**b**). Cells were incubated at 4 °C, 13 °C, 22 °C (RT) or 37 °C for the indicated times in the presence of AngII (100 nM) before BRET measurements. The BRET signals are expressed as a percentage of BRET ratios observed in the control (without AngII treatment). Data shown represent the means±s.e.m. of three independent experiments. (**c**,**d**) HEK293 cells were transfected with AT1R-RLucII/Lyn-rGFP (**c**) or AT1R-RlucII/rGFP-FYVE (**d**) along with either pcDNA or β-arrestin2. Cells were treated with the indicated concentrations of AngII for 30 min (**c**) or 40 min (**d**) before BRET measurements. Raw BRET ratios are the means±s.e.m. from at least three independent experiments. (**e**,**f**) HEK293 cells were transfected with AT1R-RlucII/rGFP-FYVE (**e**) or AT1R/βarr2-RlucII/rGFP-FYVE (**f**), along with either pcDNA or dynamin K44A (DynK44A, O). Cells were incubated in the absence (control, ▪) or in presence of 0.45 M sucrose (▴) for 20 min, and stimulated with the indicated concentrations of AngII for 40 min before BRET measurement. Raw BRET values are the means±s.e.m. of three independent experiments.

The kinetics of receptors endocytosis and trafficking to EEs were examined using AT1R-RlucII with either Lyn-rGFP or rGFP-FYVE. AT1R sequestration from the PM was faster (*t*_1/2_≈3 min) than the accumulation of receptor in EEs (*t*_1/2_≈9 min; Fig. 2d–f). Agonist-promoted sequestration of receptors, as assessed either by the disappearance of receptors from the PM or their accumulation in EE, was temperature-sensitive, being most efficient at 37°C (Fig. 3a,b). The marginal reduction in BRET response observed at the PM at 4 °C likely represents a small fraction of activated AT1R being sequestered away from the Lyn-rGFP (for example, clathrin-coated pits or other membrane compartments), but, like the remaining agonist-occupied receptors, these were unable to accumulate in EEs at such temperature. In agreement with β-arrestins' role in GPCR endocytosis^26^,^27^, overexpressing β-arrestin2 enhanced AT1R removal from the PM and their accumulation together in EEs, as determined by EbBRET (Fig. 3c,d, respectively). Similar effects were also observed with β2AR and β1-adrenergic receptors (β1AR) (Supplementary Fig. 7). DynK44A expression or treatment of cells with known endocytosis inhibitors (for example, sucrose, phenylarside oxide and concanavalin A)^31^,^32^ blocked AT1R, β1AR and β2AR sequestration from the PM, as well as the trafficking of receptors to EEs (Fig. 3e,f; Supplementary Fig. 8a–g). Inhibiting vesicle acidification with bafilomycin A or chloroquine, which block AT1R recycling^33^ from EEs to the PM, increased both the basal and agonist-promoted EbBRET signal in EEs (Supplementary Fig. 8h).

**Figure 4:**
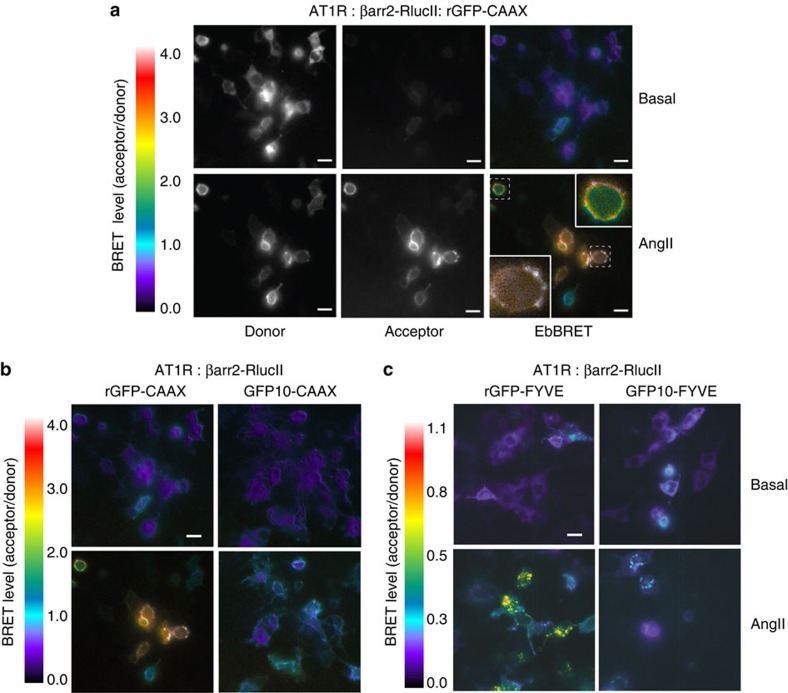
Imaging EbBRET for β-arrestin2 trafficking in response to AT1R stimulation. (**a**) HEK293 cells were transfected with AT1R, βarr2-RlucII and rGFP-CAAX. Luminescence images were acquired after the addition of the luciferase substrate, Prolume Purple coelenterazine, using the optical filters for BRET donor (370–450 nm) or acceptor (longer than 480 nm). Exposure time was 90 s for each picture. To generate BRET images, the ratio of acceptor photon counts to donor photon counts was calculated for each pixel and expressed as a color-coded heat map (lowest being black and purple, and highest red and white; see heat-map legend). Cells were treated (bottom panels) or left untreated (top panels) with 100 nM AngII for 10 min at room temperature (RT). Images show BRET signals of the same cells before and after the treatment with AngII. Insets show magnified images of cells from dashed boxes. (**b**,**c**) HEK293 cells were transfected with AT1R, βarr2-RlucII and either rGFP-CAAX or GFP10-CAAX (**b**), or with either rGFP-FYVE or GFP10-FYVE. (**c**) Luminescence images were obtained and BRET images generated after 10 min at RT (**b**) or 1 h at 37 °C (**c**) AngII (100 nM) stimulation as described in the Methods section. The numeric scale of the heat-map legend represents calculated BRET ratios. Scale bars, 20 μm.

Given the limitation of spatio-temporally resolved BRET imaging due to the low signal intensity of conventional BRET pairs^34^,^35^, we assessed whether the higher EbBRET signal observed between βarr2-RlucII and rGFP-CAAX or rGFP-FYVE improved subcellular BRET imaging both at the PM and EEs. Figure 4a,b and Supplementary Movie 1 illustrate β-arrestin2 recruitment to the PM upon AT1R stimulation. Agonist-promoted translocation of both AT1R (Supplementary Fig. 9) and β-arrestin2 (Fig. 4c) into EEs could also be readily imaged. These clear BRET images obtained with the RlucII:rGFP pairs contrast with the very weak signals obtained with the RlucII:GFP10 pair (Fig. 4b,c; Supplementary Fig. 9).

### Assessing recycling and forward trafficking of GPCRs

**Figure 5:**
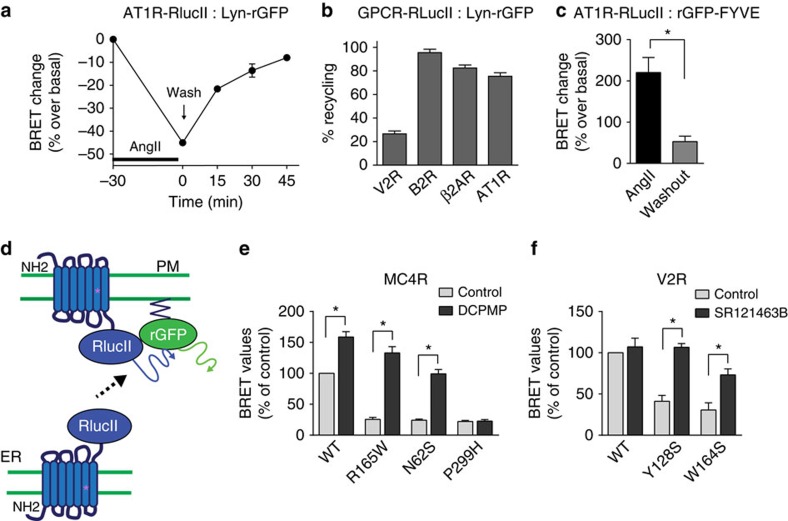
Monitoring GPCR recycling and forward trafficking of mutant receptors using EbBRET sensors. (**a**) Recycling time course of AT1R to the PM. HEK293 cells co-expressing AT1R-RLucII and Lyn-rGFP were first stimulated for 30 min with AngII (100 nM), then washed and incubated in the absence of agonist for the indicated times. The BRET signals were expressed as a per cent change over the basal (without AngII treatment, representing 0% internalization). (**b**) GPCR recycling to the PM for different receptors. HEK293 cells expressing Lyn-rGFP, along with either V2R-RlucII, B2R-RlucII, β2AR-RlucII or AT1R-RlucII were subjected to receptor recycling protocol as described in **a** after stimulation with their cognate ligands (100 nM AVP for V2R, 100 nM BK for B2R, 1 μM Iso for β2AR and 100 nM AngII for AT1R). Receptor recycling is expressed as a per cent recovery of BRET signals from internalized receptor after ligand removal as calculated in the Methods section. (**c**) Disappearance of AT1R from EEs. Cells expressing AT1R-RLucII along with rGFP-FYVE were incubated in the absence (control) or presence of 100 nM AngII for 30 min. Cells were washed and further incubated in the absence of agonist for 45 min. The BRET signal is expressed as a per cent change over the basal (without AngII, set at 0%). (**d**–**f**) Rescue of mutant receptors to the PM using EbBRET assay. (**d**) Illustration of the EbBRET assay designed to assess the PM targeting of ER-retained mutant forms of receptors by pharmacological chaperones. (**e**,**f**) HEK293 cells were co-transfected with rGFP-CAAX and either WT or mutant forms of MC4R-RlucII (**e**) or V2R-RlucII (**f**). BRET was measured in the presence or absence of 100 μM of MC4R- and V2R-selective pharmacological chaperons (DCPMP and SR121463B in **e** and **f**, respectively) for 16 h. Data are expressed as a per cent of the BRET observed for each of the WT receptors in the absence of pharmacological chaperon treatment (control). All data represent the means±s.e.m. from three independent experiments. *P<0.05, unpaired Student's *t*-test.

We next assessed receptor recycling using our EbBRET sensors. As indicated above, AngII treatment for 30 min promoted a 45% decrease in the AT1R-RlucII:Lyn-rGFP EbBRET signal, demonstrating receptor internalization from the PM. On AngII washout, 82.3±1.2% of internalized receptors recycled back to the PM in a time-dependent manner (Fig. 5a). As is the case for the AT1R, B2R and β2AR recycle back to the PM following agonist removal, whereas vasopressin type 2 receptor (V2R) recycles poorly^23^,^36^,^37^. Respectively, 95.5±2.9% and 82.5±2.5% of internalized B2R and β2AR recycled to the cell surface, while only 26.6±2.4% of internalized V2R reappeared at the PM following agonist removal (Fig. 5b). Receptor recycling was also assessed from the vantage point of receptor disappearance from EEs, using AT1R-RlucII and rGFP-FYVE. As previously observed (Figs 2 and 3), AngII treatment promoted an increase in EbBRET signal, reflecting accumulation of receptors in EEs. On AngII removal, AT1R disappeared from EEs, as seen by a 76.6±3.3% loss in EbBRET signal (Fig. 5c), which was in agreement with 82.3±1.2% of receptor recovery at the PM, observed with AT1R-RlucII:Lyn-rGFP (Fig. 5a).

In many GPCR disease-causing mutations, forward trafficking of receptors is impeded as a result of their misfolding and sequestration in the endoplasmic reticulum (ER)^6^,^7^. Examples include the V2R and melanocortin type 4 receptor (MC4R) involved in nephrogenic diabetes insipidus and early-onset obesity, respectively^38^,^39^. Because the expression at the PM of these mutant receptors can been rescued by PCs, we assessed whether our biosensors could be used to assess the activity of such drug candidates (Fig. 5d). For this purpose, EbBRET between ER-retained mutant forms of either MC4R-RlucII or V2R-RlucII, and rGFP-CAAX was determined in the presence or absence of known MC4R- (DCPMP, *N*-((2R)-3(2,4-dichlorophenyl)-1-(4-(2-((1-methoxypropan-2-ylamino)methyl)phenyl)piperazin-1-yl)-1-oxopropan-2-yl)propionamide) or V2R-selective (SR121463B) PC. As illustrated in Fig. 5e,f, significantly lower EbBRET signals were observed for the ER-retained mutant MC4R (R165W, N62S and P299H) and V2R (Y128S and W164S) as compared with their wild-type (WT) counterpart, suggesting lower levels of mutant receptors expression at the PM. Overnight treatment of cells with their respective PC restored, towards values observed for their WT counterparts, the EbBRET signals for both R165W- and N62S-MC4R (Fig. 5e), as well as Y128S- and W164S-V2R (Fig. 5f), consistent with the known ability of these mutant receptors to be rescued by their respective PC^40^,^41^,^42^ and with previous studies showing that DCPMP does not promote P299H-MC4R rescue^41^. Interestingly, PC treatment increased EbBRET between rGFP-CAAX and the WT MC4R-RlucII, but not WT V2R-RLucII (Fig. 5e,f), in agreement with the reported sensitivity of these receptors to PC actions^40^,^41^. The kinetics of PC action can be easily monitored using EbBRET biosensors, as illustrated by the time-dependent increase in EbBRET signals observed for R165W- and N62S-MC4R, following PC treatment and their reduction following PC removal (Supplementary Fig. 10, left and right panel, respectively).

### Scalability of EbBRET assays

We next determined whether these sensitive EbBRET biosensors could also be used in a high-throughput format to study protein trafficking using 96-well plates (Supplementary Fig. 11). The *Z′* factor^43^ that statistically determines the robustness and reproducibility of an assay from the dynamic range and variability of responses (for example, a *Z*′ factor of at least 0.50 being amenable for high throughput) were between 0.64 and 0.83 for all assays considered (Supplementary Fig. 11a,b). The *Z*′ factors obtained for the RlucII:rGFP pairs, for all biosensors tested, where far superior to those obtained for the RlucII:GFP10 combination (that varied between negative values and 0.4; Supplementary Fig. 11c). To illustrate the usefulness of the biosensors in true high-throughput format (for example, in 384-well plates), we performed a limited screening campaign with AT1R-RlucII and rGFP-FYVE using a commercial library (containing ∼1200 compounds, which included AT1R antagonists of the ‘Sartan' family). The *Z*′ factor, in a fully automated 384-well plates format, was 0.65±0.02 (*n*=3) (Supplementary Fig. 11d; Supplementary Data 2). In an unbiased manner, we identified five Sartans in the library, which significantly inhibited the trafficking of AT1R to EEs (Supplementary Fig. 11e). These results support the scalability and usefulness of EbBRET biosensors for high-throughput screens.

### Biased intracellular sorting of AT1R revealed by EbBRET

**Figure 6:**
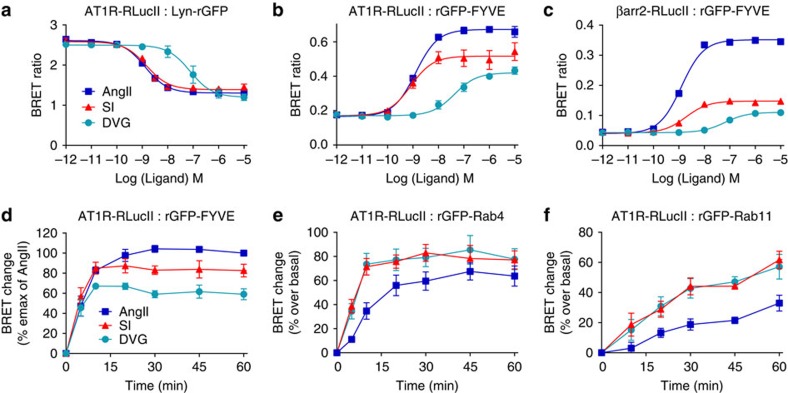
Differential effects of AngII analogues on AT1R trafficking and sorting, as assessed by EbBRET. HEK293 cells were transfected with AT1R-RlucII/Lyn-rGFP (**a**), AT1R-RlucII/rGFP-FYVE (**b**), or AT1R/βarr2-RlucII/rGFP-FYVE (**c**), and incubated with the indicated concentrations of AngII (blue), SI (red), or DVG (turquoise) for 30 min (**a**) or 40 min (**b**,**c**) before BRET measurements. Data are represented as the means±s.e.m. from at least three independent experiments. (**d**) HEK293 cells expressing AT1R-RLucII and rGFP-FYVE were incubated either with 100 nM AngII (blue), 100 nM SI (red) or 1 μM DVG (turquoise) for indicated times before BRET measurements. The agonist-mediated BRET ratio changes were normalized to the maximal AngII response (60 min), which was set as 100% and the basal (without ligand) was set as 0%. Data are expressed as the means±s.e.m. of three independent experiments. (**e**,**f**) HEK293 cells were transfected with 120 ng of AT1R-RlucII and with either 360 ng of rGFP-Rab4 (**e**) or 270 ng of rGFP-Rab11 (**f**). Cells were incubated with either 100 nM AngII (blue), 100 nM SI (red) or 1 μM DVG (turquoise) for the indicated times before BRET measurements. Ligand-mediated BRET changes are expressed as per cent changes over the basal (without ligand, set at 0%). Data represent the mean±s.e.m. from three independent experiments.

AngII analogues have been shown to have distinct biased signalling properties^4^ and proposed to promote differential trafficking of the receptor^15^. Thus, we examined whether we could use EbBRET to detect such bias in receptor trafficking. We compared the potency and efficacy of the AngII analogues SI and DVG to promote AT1R sequestration from the PM and receptor accumulation in EEs. SI and DVG are two selective β-arrestin-biased AngII ligands (that is, not promoting Gq activation) that show high affinity for AT1R^3^,^4^,^41^,^42^,^43^ (for example, SI: 1–7 nM (refs 4, 44, 45, 46) and DVG: 17 nM). As shown previously, they both promote AT1R internalization and stable receptor/β-arrestin complexes in EEs^3^,^4^. However, the complex dissociation in EEs was shown to be faster than the one observed with AngII following agonist removal, suggesting that the avidity between AT1R and β-arrestin, in the case of SI and DVG occupied receptors, is weaker than for AngII, and that the avidity of this complex doesn't necessarily correlate with the affinities of ligands^3^,^4^. In the EbBRET assay, AngII, SI and DVG decreased the signals at the PM in a concentration-dependent manner with similar efficacies (Fig. 6a). SI and AngII showed similarly higher potencies to promote AT1R disappearance from PM than DVG (EC_50_ of 1.3, 1.5 and 75 nM for AngII, SI and DVG, respectively). Both SI and DVG were less efficacious at promoting AT1R and β-arrestin2 accumulation in EEs (Fig. 6b,c), suggesting that AngII analogues cause distinct sorting of AT1R/β-arrestin2 complexes—perhaps to different cellular compartments. Thus, we compared the accumulation of receptors in EEs promoted by different ligands over time. At concentrations of ligands occupying >90% of receptors, DVG- and SI-bound AT1R reached maximal accumulation in EEs after 10 min, while AngII only attained this level after 20 min. However, maximal accumulation of receptors in EEs promoted by DVG and SI were 40 and 20% less than with AngII, respectively (Fig. 6d), consistent with a ligand-biased sorting of receptors. To test the potential differential sorting of AT1R by SI or DVG, which may explain the lower accumulation of receptors in EEs, we generated additional EbBRET-based sensors. Because AT1R has been shown to traffic through sorting and recycling endosomes^47^, we tagged Rab4 and Rab11 with rGFP. Both rGFP-Rab4 and rGFP-Rab11 showed vesicular localizations that were largely distinct from Rab5-containing EEs (Supplementary Fig. 12). EbBRET revealed that AngII-promoted AT1R-RlucII accumulation in Rab4-containing vesicles was faster than for Rab11-containing endosomes, in agreement with previous findings^48^,^49^. Over time, SI and DVG stimulation of AT1R generated faster and higher EbBRET signals with rGFP-Rab4 and rGFP-Rab11 than AngII stimulation (Fig. 6e,f), which most likely results from a faster sorting of AT1R from EEs to sorting and recycling endosomes when stimulated with SI or DVG.

## Discussion

BRET requires the physical proximity of a donor and an acceptor, which is imparted by interacting proteins fused respectively to these chromophores^50^,^51^. BRET signals can also emanate from random collisions (also known as bystander BRET), when chromophores are present in close proximity and in sufficiently high concentrations relative to each other, such as when proteins co-localize in the same bidimensional membrane compartment^50^. The EbBRET approach using the RlucII:rGFP pair described here takes advantage of this latter property and is distinct from classical BRET assays, as it is specifically designed to monitor the translocation to, or from a given compartment by selectively targeting the energy acceptor to an organelle and not to a specific protein complex. The *Renilla reniformis* chromophore pair Rluc and rGFP has been used to improve the sensitivity of BRET signal for monitoring protein–protein interactions^10^,^19^ and to study receptors removal (that is, internalization) from PM^19^. Here we devised new sets of EbBRET trafficking sensors, which not only improved the quantitative monitoring in real time of the loss of PM receptor following agonist stimulation (that is, a decrease in BRET signal through sequestering RlucII-tagged receptors away from rGFP anchored at the PM), but also allow monitoring the trafficking of receptors and β-arrestin to EEs (that is, an increase in BRET signal from the accumulation of RlucII-tagged receptors or β-arrestin with rGFP anchored in EEs), as well as to assess GPCR activity (for example, translocation of β arr2-RlucII to rGFP anchored at the PM). Targeting rGFP to distinct endosomal compartments using selectively localized Rab proteins also permitted to follow the progression of receptors in these compartments. These sensors therefore allow studying, with high sensitivity, the complete internalization/recycling cycle of receptors, as well as their forward trafficking, and are amenable to high-throughput screens for identifying modulators of receptor trafficking. Recruitment of β-arrestin to the PM and its localization to EEs was quantitatively monitored, both spectrometrically and by microscopy BRET imaging using βarr2-RlucII with either PM or EEs rGFP-tagged markers. The recycling of receptors from EEs to the PM following stimulus removal, as well as the rescue of ER-retained GPCR mutants by PC, and ligand-mediated biased trafficking was also readily measured in real time. In all cases, our sensors captured the expected trafficking behaviours of receptors and β-arrestin.

The non-natural BRET pairs (for example, combination of Rluc from *Renilla reniformis* with GFP variants from *Aequorea victoria*) have been employed with success to investigate the subcellular distribution of proteins, including β-arrestin recruitment, receptor internalization, and endosomal and Golgi trafficking, and for monitoring PC action at the ER and PM^3^,^12^,^13^,^14^,^15^,^16^. Direct comparison between rGFP and GFP10-based sensors (Fig. 2; Supplementary Fig. 5) clearly shows that the magnitude of the signal and, in most instances, the dynamic windows are greater for the natural RlucII:rGFP BRET pair. Such sensitivity greatly facilitates the study of protein kinesis both spectrometrically and by microscopy, in addition to allow HTS. Indeed, we observed significantly better *Z*′ factors when using rGFP versus GFP10 in scaled-up assays. The rGFP-based sensor was also more sensitive to detect endogenous GPCR activation (for example, β-arrestin2 translocation) and responses to partial agonist stimulation of receptors with lower avidity for β-arrestin like in the case of the β2AR^20^. The lower-energy transfer efficiency between RlucII and GFP10 or GFP2—classically used in BRET2 experiments—as opposed to RlucII:rGFP, which spontaneously interact together and have evolved to adopt optimal conformations for energy transfer^52^, likely explains the reduced sensitivity, in some cases, of the non-natural chromophores. Advantageously, the RlucII:rGFP interaction does not preclude the RlucII- and rGFP-tagged proteins from separating when trafficking to, or from different cellular compartments, thus, allowing real-time examination of GPCR/β-arrestin-internalization-trafficking-recycling life cycles. Similar dissociation properties between Rluc and rGFP were also reported in a self-interacting chimeric protein upon cleavage, supporting the reversibility of the chromophores' interactions in cells^10^. Because rGFP has been reported to self-dimerize, caution should also be taken when tagging proteins with this chromophore^53^, since it may change their properties. Most likely this had little impact on our acceptor biosensors, since the rGFPs were not attached to the proteins under study, but rather targeted to the PM or in EEs using small targeting motifs, (for example, Lyn-rGFP and rGFP-FYVE). We verified the proper localization of the acceptor biosensor and the dimerization of rGFP did not prevent their proper distribution. A free N terminus in rGFP was previously reported to be necessary for high-efficiency BRET transfer^10^. Consistent with the lower efficiency of a N-terminal-tagged rGFP to act as a BRET acceptor, we observed much greater BRET signals when using the C-terminally tagged rGFP-CAAX versus the N-terminally tagged Lyn-rGFP (Supplementary Fig. 5).

BRET imaging has been previously used to study protein interactions, and protein distribution in cells and *in vivo*^34^,^35^,^54^,^55^. However, the long acquisition time required due to low-intensity signals limited high-resolution subcellular compartmental examination in cells. The use of RlucII:rGFP pairing, combined with a long-lasting Rluc substrate (that is, Prolume Purple coelenterazine) and a sensitive detection system, has allowed for real-time video imaging of EbBRET signal of β-arrestin2 translocation to the PM for up to 20 min. Importantly, we were able to detect with high-resolution clustering of receptor/β-arrestin2 complexes at the PM and in EEs, at microscopic detection levels that was not previously achieved with other BRET imaging approaches. EbBRET-imaging prevents photobleaching/phototoxicity and background signals because no external excitation light sources are used, and little cross-bleeding from the donor into the acceptor channel is observed, which often leads to artefacts in fluorescence resonance energy transfer analysis. Thus, EbBRET-imaging represents an important advancement towards quantitative visualization of proteins' behaviour within different subcellular domains in real time without the limitations imposed by other florescence microscopy approaches.

EbBRET provides the ability to study, with high sensitivity, pathways of endocytosis, and the ability of different drugs to promote distinct path and kinetics of endosomal GPCR trafficking, which has been previously difficult to examine by other conventional assays (for example, ligand binding, FACS and so on). Indeed, our use of EbBRET revealed the ligands' propensity to differentially control the trafficking of receptor/β-arrestin complexes to EEs versus recycling endosomes. The reproducibility and robustness of such approach should allow the use of these sensors in HTS to identify modulators of receptor/β-arrestin trafficking and for other agents, such as PC, capable of rescuing receptor mutants associated with diseases.

The realization that biosensors based on the highly efficient RLuc:rGFP BRET pair can be used to assess not only receptor trafficking in live cells, but also the activity of adaptor proteins like β-arrestin, opens new opportunities for engineering novel and more sensitive EbBRET-based sensors for other effectors requiring re-localization within the cells for their activity. The approaches and sensors presented here, therefore, provide a versatile platform to study the trafficking of GPCRs and other classes of proteins, including channels, transporters and enzymes, making it a universal assay to monitor and image protein kinesis in cells.

## Methods

### Materials

AngII, poly-L-ornithine, arginine–vasopressin, Bradykinin and Isoproterenol were from Sigma. Prostaglandin F2α was from Cayman Chemical (Ann Arbor, MI). [Sar^1^, Ile^8^]-AngII (SI) and [Asp^1^, Val^5^, Gly^8^]-AngII (DVG) were synthesized at the Université de Sherbrooke (Canada, QC). SR121463B was kindly provided by Sanofi Aventis (Bridgewater, NJ). DCPMP was synthetized at the medicinal chemistry platform of the Institute for Research in Immunology and Cancer (IRIC, Montreal, Canada). SLIGKV-NH_2_ and salbutamol were from Tocris Bioscience (Bristol, United Kingdom). Iodine-125 was obtained from PerkinElmer. Dulbecco's modified Eagles medium (DMEM), fetal bovine serum (FBS) and other cell culture reagents were purchased from Invitrogen. Prolume Purple (Methoxy e-CTZ), coelenterazine 400a and coelenterazine-h were purchased from Nanolight Technology and Goldbio. Phusion DNA polymerase was from Thermo Scientific. Restriction enzymes and T4 DNA ligase were obtained from NEB. HTS was performed at the IRIC (Université de Montreal) using 1,260 compounds from Microsource Discovery Spectrum library (msdiscovery.com).

### Plasmids and constructions

For the construction of the Lyn-GFP10, the coding sequence of the first 11 residues (MGCIKSKGKDS) of the human Lyn-kinase and the full coding region of GFP10^56^ (Supplementary Data 1) were synthesized at GenScript (Piscataway, NJ) and subcloned into pcDNA 3.1/zeo (−) using infusion (Clontech, CA). A humanized rGFP (Supplementary Data 1) was synthetized at GenScript (Piscataway, NJ). The Lyn-rGFP was generated by replacing the coding sequence of GFP10 in the Lyn-GFP10 construct by rGFP, which was generated by PCR amplification. StreptagII-fused GFP10 was synthesized at GenScript and subcloned into pcDNA 3.1/zeo (−) (STII-GFP10). The DNA linker for CAAX motif, a PM-targeting polybasic sequence and prenylation signal sequence from K-RAS splice variant b (KKKKSKTKCVIM), was inserted into BamHI and HindIII sites of STII-GFP10 in-frame by DNA ligation (GFP10-CAAX). Plasmid encoding the fusion protein rGFP-CAAX was obtained by PCR amplification of rGFP-coding sequence with a reverse primer encoding a linker (GSAGTMASNNTASG) and the PM-targeting sequence: GKKKKKKSKTKCVIM. The CAAX-targeting sequence is in-frame at the C terminus of the rGFP-coding sequence. The PCR fragment was subcloned in the NheI and EcoRI sites of pcDNA3.1 (+) vector. The FYVE domain of the human endofin (residues from Q739 to K806) was synthesized at Bio Basic Inc. (Ontario, Canada) and subcloned into the STII-GFP10 construct in-frame (GFP10-FYVE). rGFP-FYVE was generated by inserting the FYVE domain of GFP10-FYVE into a vector containing humanized codons for rGFP in pcDNA3.1(+) in-frame. rGFP-Rab4 and rGFP-Rab11 were generated by replacing the FYVE domain in rGFP-FYVE with PCR-amplified Rab4 and Rab11-coding sequences, respectively. To generate RlucII-fused AT1R, the human AT1R-coding sequences containing a signal peptide and the FLAG sequence were amplified by PCR, and subcloned in-frame into pcDNA3.1/hygro(+) also containing the RlucII using the NheI and HindIII sites. Plasmids encoding human βarr2-RlucII have been previously described^57^. To generate the different MC4R-RlucII constructs, coding region of hMC4R-WT, hMC4R-(R165Q) or hMC4R-(P299H) were PCR amplified and products were subcloned in-frame at the N terminus to the RlucII sequence^20^ (Supplementary Data 1) into pcDNA3.1 RlucII vector using the linker sequence; VGGGGSKLPAT. The V2R substitution Y128S was created using the site-directed mutagenesis with the Quick Change mutation kit (Agilent Technologies, Santa-Clara, USA) from the human V2R (hV2R) complementary DNA (hV2R-Y128S). Plasmids encoding the fusion protein hV2R WT-RlucII and hV2R (Y128S)-RlucII were obtained by PCR amplification of hV2R-coding sequence and subcloned in-frame at the N terminus to the RlucII sequence into pcDNA3.1 RlucII vector (linker sequence: GGSGLKLPAT). All the PCR were done by using the Phusion DNA polymerase and constructs were verified by DNA sequencing before use.

### Cell culture and transfection

HEK293SL (ref. 58; referred in the result section as to HEK293 cells), which are a subclone derived from regular HEK293 cells (Ad5 transformed) and selected in our lab, have been used in all experiments. These cells have a cobblestone appearance, and show better adherence as compared with regular HEK293 and HEK293T cells, making them more amenable to microscopy and BRET experiments. These cells were regularly tested for mycoplasma contamination (PCR Mycoplasma Detection kit, abm, BC, Canada) and cultured in DMEM supplemented with 6% FBS and 20 μg ml^−1^ of gentamycin. Cells were grown at 37 °C in 5% CO_2_ and 90% humidity. Cells were transfected either by calcium phosphate or polyethylenimine (PEI) methods. For calcium phosphate transfection, cells were seeded at a density of ∼7.5 × 10^5^ per 100-mm dish 1 day before transfection, and transfection was carried out as described previously^59^. After 18 h of transfection, the medium was replaced, and cells were divided for subsequent experiments. For PEI transfection, HEK293 cells were seeded directly into 96-well plates (35,000 cells per well) and transfected simultaneously as described previously^60^. All assays were performed 48 h after transfection.

### BRET measurements

HEK293 cells were transfected with a BRET donor (120 ng of Receptor-RlucII or 72 ng of βarr2-RlucII) along with 480 ng of BRET acceptor (for example, Lyn-rGFP, rGFP-FYVE or rGFP-CAAX) otherwise the DNA amount was specified in the Methods section and/or the figure legends. The day following transfection, cells were detached and re-seeded onto poly-L-ornithine coated, white, 96-well plates at a density of ∼25,000 cells per well. The next day, cells were washed once with pre-warmed Tyrode's buffer (140 mM NaCl, 2.7 mM KCl, 1 mM CaCl_2_, 12 mM NaHCO_3_, 5.6 mM D-glucose, 0.5 mM MgCl_2_, 0.37 mM NaH_2_PO_4_, 25 mM HEPES and pH 7.4), and then stimulated with either various concentrations of ligand in Tyrode's buffer for 30–40 min, or a single concentration of ligand for various times at 37 °C. For recycling experiments, ligand-simulated cells (for 30 min at 37 °C) were washed either four times with ice-cold Tyrode's buffer or three times with acid (50 mM sodium citrate, pH 4.0), followed by washing two times with Tyrode's buffer. All washing steps were performed on ice. Cells were then further incubated with Tyrode's buffer at 37 °C for 45 min or indicated times before BRET measurements. The cell-permeable substrate, coelenterazine 400a or Prolume Purple (final concentrations of 5 and 1.3 μM, respectively) was added 3∼6 min before BRET measurements. For evaluation of cell surface expression rescue by PCs, HEK293 cells were seeded and transfected in 96-well plates using PEI. The DNA transfected per well of a 96-well plate is, for V2R: 1.2 ng of hV2R-RlucII and 4.8 ng of rGFP-CAAX, and for MC4R, 2.4 ng of hMC4R-RlucII and 7.2 ng of rGFP-CAAX. Twenty-four hours post transfection, cells were treated either with PC (for MC4R: 10 μM DCPMP; for V2R: 10 μM SR121463b) or dimethylsulphoxide (DMSO, as control conditions). After 16–18 h of PC treatment, cells were washed once with phosphate-buffered salins (PBS) and left in Tyrode's buffer. The Rluc substrate, coelenterazine 400a (for BRET2 MC4R experiments) or coelenterazine-h (for BRET1 V2R experiments) was added at a final concentration of 2.5 μM and cells were further incubated for an additional 5 or 10 min, respectively, before BRET measurements. For measurement of BRET in HEK293 cells with the β1AR, 10 nM of ICI118.551 was used for 30 min before receptor stimulation to block endogenous β2AR. All the BRET measurements were performed using either a Synergy2 (BioTek) microplate reader with a filter set (centre wavelength/band width) of 410/80 nm (donor) and 515/30 nm (acceptor), or using a Mithras LB940 Multimode Microplate Reader, equipped with the following filters for BRET2: 400/70 nm (donor) and 515/20 nm (acceptor), and for BRET1: 480/20 nm (donor) and 530/20 nm (acceptor), for detecting the RlucII *Renilla* luciferase (donor) and GFP10 or rGFP (acceptor) light emissions, respectively. Raw BRET ratio was determined by calculating the ratio of the light intensity emitted by the GFP10 or rGFP over the light intensity emitted by the RlucII. The BRET signals are normalized to basal BRET ratios (without ligand) and expressed as a per cent basal ((BRET_ligand_/BRET_basal_) × 100), so that the basal BRET is set at 100%. Agonist-promoted BRET changes were expressed as a per cent over basal (((BRET_ligand_−BRET_basal_)/BRET_basal_) × 100), so that basal is set at 0%. Receptor recycling (% recycling) was calculated as follows: ((BRET signal_ligand washout_−BRET signal_ligand_)/(BRET signal_basal_−BRET signal_ligand_) × 100), where BRET signal_ligand washout_ represents the BRET signals after ligand removal (for example, 45 min) and BRET signal_ligand_ represents the BRET signals after ligand stimulation (30 min, representing maximal internalization).

### Luminescence spectrum measurement

HEK293 cells were cultured in DMEM supplemented with 10% FBS, 100 units per ml penicillin and 0.1 mg ml^−1^ streptomycin and transfected with 100 ng of RLucII N-terminally fused to prototypical acceptors for BRET1 (that is, venus) and BRET2 (that is, GFP2^56^, sequence provided in Supplementary Data 1) or to rGFP per well in a six-well plate. GFP10 and GFP2 share similar spectral properties as BRET2 acceptors^56^. Transfections were done with a ratio of 1 μg DNA: 3 μl X-treme GENE HP reagent (Roche), according to the manufacturer's protocol. Cells were detached from the culture surface by adding 1 ml of PBS supplemented with 5 mM EDTA, and re-suspended in PBS. One μM of Prolume Purple (Nanolight Technology) was added and the luminescence spectrum was obtained with the Synergy Neo plate reader (BioTek) 2 min following substrate addition.

### Luminescence microscopy (BRET imaging)

Cells seeded on poly-D-lysine-coated glass-bottom 35-mm culture dishes at the density of 1–2 × 10^5^ cells per dish were transfected with 100 ng per dish of AT1R, 50 ng per dish of βarr2-RlucII, and either 500 ng per dish of rGFP-CAAX or rGFP-FYVE, using X-treme GENE HP reagent (Roche). Two days post transfection, cells were washed once with 1 ml of modified Hank's balanced salt solution (138 mM NaCl, 5.33 mM KCl, 0.44 mM KH_2_PO_4_, 0.33 mM Na_2_HPO_4_, 4.16 mM NaHCO_3_, 1.0 mM CaCl_2_, 1.0 mM MgCl_2_, 10 mM HEPES, pH 7.4) and set on the microscope. BRET images were obtained using Nikon Ti-U microscope equipped with × 60 objective (Apochromat TIRF, NA 1.49, Nikon) and imaging camera (PIXIS1024, Princeton instruments) with filter changer (Lambda 10-2, Sutter instrument). Immediately after the addition of Prolume Purple (final concentration of 10 μM), the camera shutter was closed and a blank image was acquired for 90 s. For BRET image intensity analysis, signals were acquired without filter and with filters corresponding to the BRET donor (370–450 nm) and BRET acceptor (480LP) wavelength for 90 s each. Images were captured every 5 min, and blank image values were subtracted from the corresponding pixels of luminescence images to remove photon counts deriving from dark current and sampling noises of the camera. For each time points, BRET ratio images were generated using pixel arithmetic functions of MetaMorph software version 7.8 (Molecular Devices) as follows: Pixel hue: BRET level calculated by dividing the counts of acceptor images with donor images, and allocated to default rainbow hue (lowest (typically 0.0) in purple and highest (typically 2.0) in red); pixel brightness: the value of donor images with auto brightness. Video images were acquired by obtaining 20 s exposure images every 30 s with BRET donor and acceptor filters. BRET donor and acceptor images were taken from two different culture dishes transfected on the same day.

### High-throughput screening

A high-throughput chemical screen using AT1R-RlucII and rGFP-FYVE was carried out at IRIC's HTS facility (University of Montreal). HEK293 cells were seeded in double CellStacks (Corning), and once they reached 60–70% confluence they were transfected with PEI (4 μg AT1R-RlucII DNA, 9 μg rGFP-FYVE DNA, 121.95 μg pcDNA, and 270 μg PEI), and grown overnight. Cells were collected and re-suspended at 1.5 × 10^6^ cells per ml in phenol red-free DMEM (Wisent) supplemented with 1% FBS and antibiotics. Using a Multidrop Combi (Thermo), 30 μl of cells were dispensed to a 384-well white tissue culture-treated plate (Greiner) and grown for an additional 24 h. On the day of screening, cells were transferred to an automated CO_2_ incubator (Thermo Cytomat). A total of 1,260 compounds from Microsource Discovery Spectrum Collections were added using automatic 384 magnetic pintool (V&P Scientific) at a final concentration of 15 μM or 5 μg ml^−1^ depending on the compound sub-library. For the antagonist mode, compounds were incubated for 30 min at 37 °C. AngII was then added at 10 nM (EC_80_) and incubated for an additional 30 min at 37 °C. rGFP fluorescence was detected using an Envision (PerkinElmer) and coelenterazine 400a was added at a final concentration of 5 μM using a multidrop 384 (Thermo). Cells were incubated at room temperature before reading the BRET signal using A SpectraMax L (Molecular Devices) with a filter set (center wavelength/band width) of 400/20 nm (donor) and 510/20 nm (acceptor).

### Radioligand binding assay

[^125^I]-AngII was prepared with the iodogen method, and specific activity was determined from self-displacement and saturation experiments, as previously described^4^. The density of cell surface receptors was evaluated with binding assays at 4 °C using [^125^I]-AngII as the tracer. HEK293 cells expressing either AT1R or AT1R-RlucII were seeded 1 day after transfection at a density of ∼120,000 cells per well in poly-L-ornithine-coated 24-well plates. The following day, cells were washed once with pre-warmed DMEM with 20 mM HEPES (DMEM-H) and then incubated in the absence or presence of 100 nM AngII in DMEM-H for 30 min at 37 °C. The plates were quickly washed three times with ice-cold acid (50 mM sodium citrate, pH 4.0) for 5 min each on ice to stop the stimulation and remove both the remaining surface bound and unbound AngII ligand. To remove and neutralize the residual acid, cells were further washed twice with ice-cold Tyrode's buffer. Cells were incubated with 0.5 ml of [^125^I]-AngII (∼250,000 c.p.m. at ∼2000, ci mmol^−1^) in binding buffer (0.2% BSA, 50 mM Tris, 100 mM NaCl_2_, 5 mM MgCl_2_, pH 7.4) at 4 °C overnight. Non-specific binding was determined in the presence of 1 μM AngII. The next day, cells were washed three times with ice-cold PBS with calcium and magnesium, and 0.5 ml of 0.5 M NaOH/0.05% SDS was added. Radioactivity from solubilized cells was counted using a PerkinElmer Wizard 1470 automatic γ-counter.

### Confocal microscopy

One day before transfection, HEK293 cells were seeded in 35-mm glass-bottom dishes at a density of 100,000 cells per dish. Cells were transfected with 83 ng of B2R-CFP, 83 ng of Lyn-GFP10 and 83 ng of mCherry-FYVE. Forty-eight hours post transfection, cells were serum starved for 30 min, and either left untreated or treated with BK (1 μM) for 15 min. Samples were analysed on Zeiss LSM-510 laser scanning microscopes using argon (514 nm) and HeNe I (543 nm) lasers, and images (2048 × 2048 pixels) were collected using a 63 × oil immersion lens. For detecting CFP and GFP, UV and argon lasers were used with 405 and 514 nm excitation, with either BP 420–480 nm or BP 505–550 nm emission filters, respectively. For mCherry detection, the HeNe I laser was used with 543 nm excitation and LP 560 nm emission filter sets. For validation of rGFP-tagged BRET acceptors, samples were analysed on the Zeiss LSM-780 laser scanning microscope with 34-channel spectral R/FL detectors (GaAsp detector array). rGFP was detected with an argon laser at 488 nm and mCherry was detected with the diode-pumped solid state (DPSS) laser (561 nm). Images (1024 × 1024 pixels) were collected using a 63 × /1.40 oil differential interference contrast (DIC) plan-apochromat lens.

### Data analysis and statistics

Estimation of the *t*_1/2_ and the EC_50_ values for ligand-mediated endocytosis were calculated using the GraphPad Prism curve fit program. The curves presented throughout this study represent the best fits and were generated using this GraphPad Prism software. Statistical analysis was performed using either Student's *t*-test two-sided or one-way analysis of variance with Turkey and Holm Sudak's post-test assuming similar variance between groups, where indicated; *P* values <0.05 were considered significant.

### Data availability

The authors declare that all data supporting the findings in this study are presented within the article and its Supplementary Information files. Sequences for RlucII, rGFP, GFP10 and GFP2 are provided in the Supplementary Data 1, and raw data of HTS for generated Supplementary Fig. 11e are provided in Supplementary Data 2. Information about HTS compounds can be obtained on request to the corresponding authors. All the biosensors generated in this study can be obtained and used without limitations for non-commercial purpose with a standard academic materials transfer agreement (MTA) on request.

## Additional information

**How to cite this article:** Namkung, Y. *et al*. Monitoring G protein-coupled receptor and β-arrestin trafficking in live cells using enhanced bystander BRET. *Nat. Commun.* 7:12178 doi: 10.1038/ncomms12178 (2016).

## Supplementary Material

Supplementary Information Supplementary Figures 1-12

Supplementary Data 1 Sequences for RlucII, rGFP, GFP10 and GFP2

Supplementary Data 2 Dataset of 1260 compounds from the HTS campaign: HEK293 cells were transfected with AT1R-RlucII and rGFP-FYVE and plated in 384-well plate for the screening as described in the Methods section. Column 1 and 2 represent plate number (plate ID) and the well treatment (well ID), respectively. Column 3 is the fluorescent of rGFP. Column 4 and 5 are the light emission from the acceptor channel (510/20 nm) and the donor channel (400/20 nm), respectively. Column 6 shows the BRET ratio which was calculated by dividing column 4 by column 5.

Supplementary Movie 1 HEK293 cells co-expressing rGFP-CAAX and βarr2-RlucII were stimulated or not with 100 nM AngII. The change of the cells morphology (e.g. shrinking) reveals its response to the agonist. Luminescence was captured as indicated in the materials and methods section following prolume purple coelenterazine using the BRET donor filter (370 nm - 450 nm) for the left panel and the and BRET acceptor filter (480LP) for the right panel. The video was generated by capturing images for 20 s every 30 s for a period of 20 min.
