# Supplementary material for: Monitoring G protein-coupled receptor and β-arrestin trafficking in live cells using enhanced bystander BRET
Source: Nat Commun. 2016 Jul 11;7:12178. doi: 10.1038/ncomms12178 (PMC4942582; doi:10.1038/ncomms12178)
Supplement: Supplementary Information — Supplementary Figures 1-12 [file ncomms12178-s1.pdf]

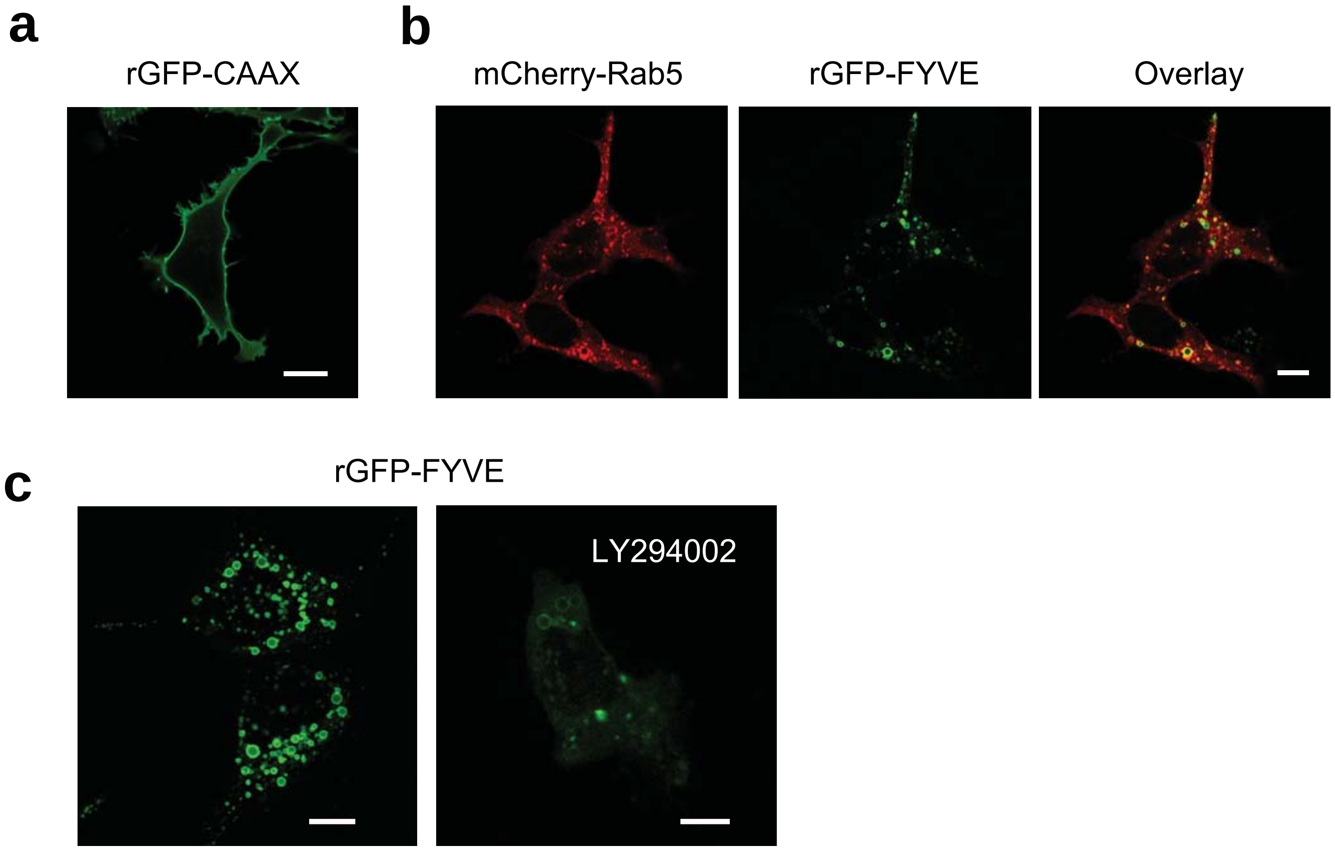

Supplementary Figure 1. Cellular localization of rGFP-CAAX, rGFP-FYVE, and mCherry-Rab5. HEK293 cells were transfected either with rGFP-CAAX (**a**), mCherry-Rab5 along with rGFP-FYVE (**b**), or rGFP-FYVE alone (**c**). Cells expressing rGFP-FYVE were treated with DMSO (left) or LY294002 (10 μM, right) for 60 min (**c**). LY294002-treated cells show progressive delocalization of rGFP-FYVE from the EE to the cytosol. Scale bars, 10 μm.

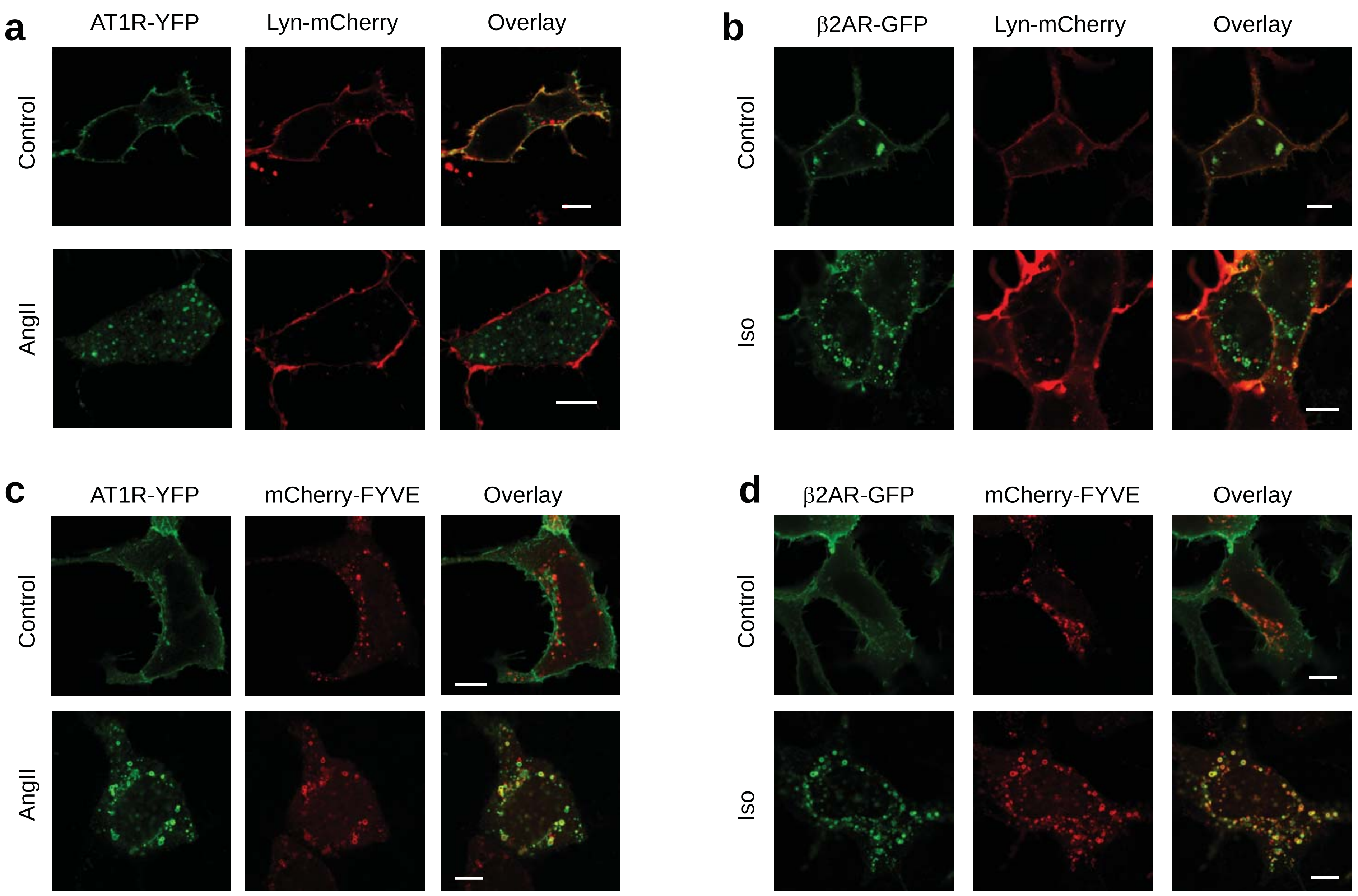

Supplementary Figure 2. Visualization of AT1R and β2AR trafficking upon receptor activation with sensors localization. HEK293 cells were transfected either with 30 ng of AT1R-YFP (**a**,**c**) or 30 ng of β2AR-GFP (**b**,**d**) along with 67 ng of Lyn-mCherry (**a**, **b**) or 67 ng of mCherry-FYVE (**c**, **d**). Images before agonist stimulation are shown as control. Cells were stimulated either AngII (final concentration of 100 nM) for ~30 min (**a**) and ~20 min (**c**) or Iso (final concentration of 10 μM) for ~10 min (**b**) and 15 min (**d**) at 37 °C. Scale bars, 10 μm.

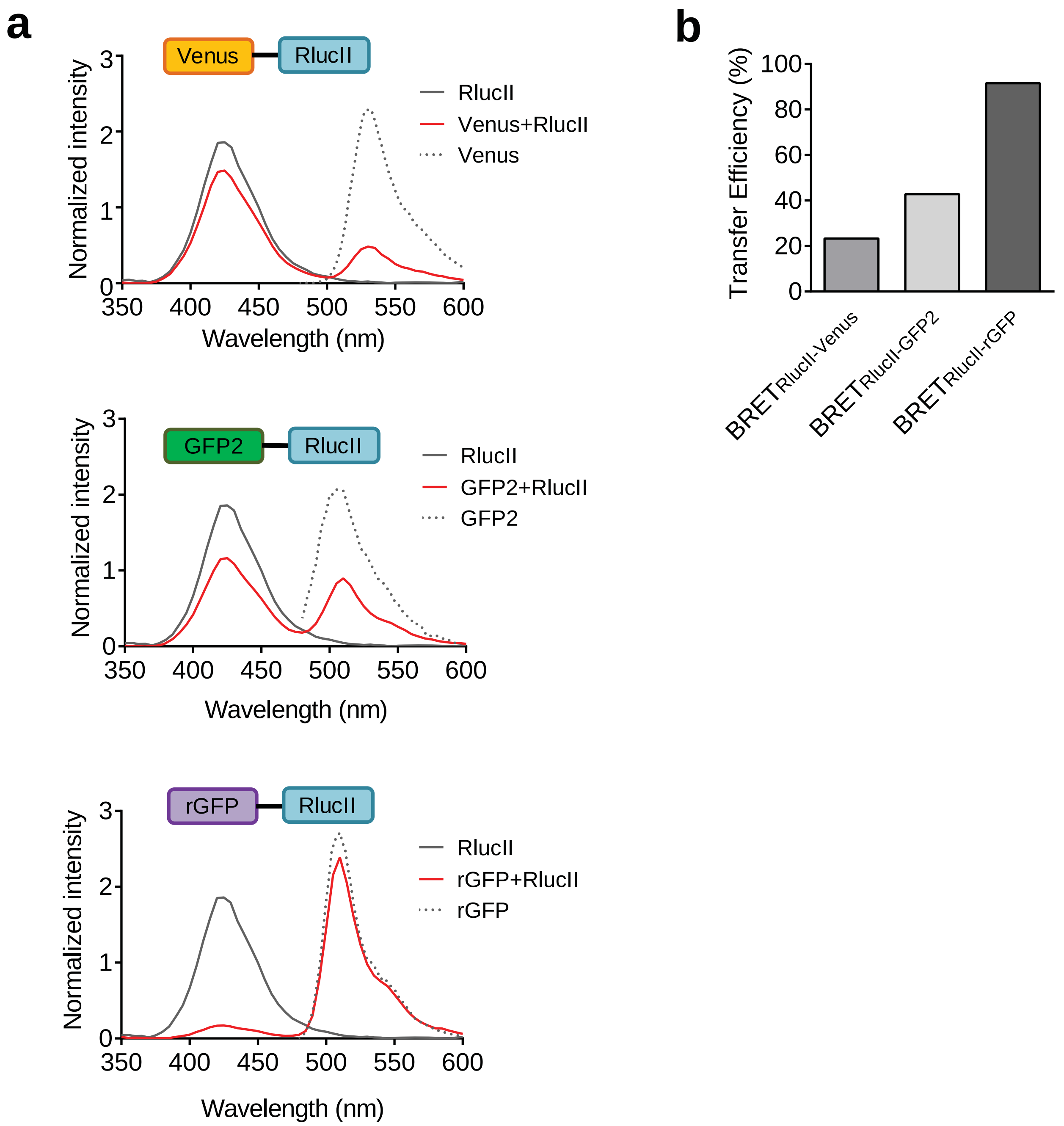

Supplementary Figure 3. Energy transfer efficiency between the different BRET pairs. (**a**) For BRET measurements, HEK293 cells were transfected with either RlucII alone or a fusion protein between RlucII and either Venus-YFP, GFP2, or rGFP, as indicated. For the fusion proteins, the C-termini of the GFPs were fused to the N-terminus of RlucII through a 19 amino acids linker. The luminescence emission was measured, as described in the Methods section, after the addition of luciferase substrate (Prolume Purple coelenterazine). The emission spectra of RlucII alone are indicated by the solid black lines, whereas emissions by the fusion proteins appear in red. The dotted line indicates the theoretical emission at the wavelengths corresponding to the GFPs' emission for 100% transfer efficiency. The emission spectra were normalized to the area under the curve obtained for the RlucII alone so that the total surface under the curves was equal for the RlucII alone and the fusion proteins. (**b**) Efficiency transfer calculated for the individual fusion constructs derived from the data presented in panel a. The data shown are representative illustration of at least 2 independent experiments.

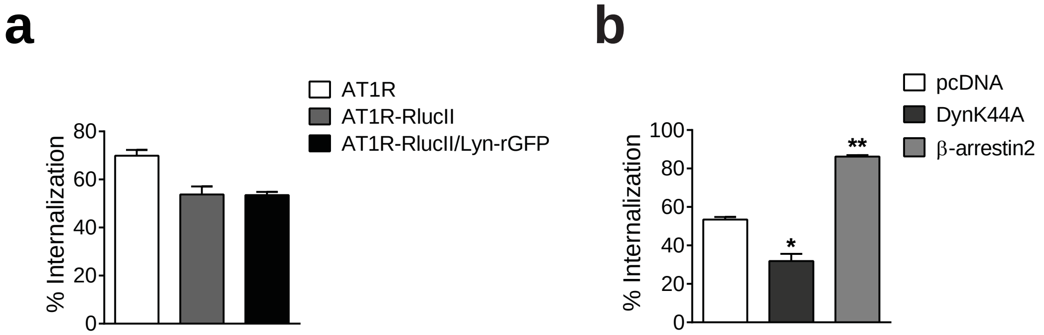

Supplementary Figure 4. AT1R internalization assessed by radioligand binding assay. (**a**) HEK293 cells were transiently transfected with 120 ng of either cDNAs for expressing AT1R alone (□), AT1R-RlucII alone (■), or AT1R-RLucII along with 480 ng of Lyn-rGFP (■). (**b**) HEK293 cells were transfected with AT1R-RlucII and Lyn-rGFP along with either pcDNA (□), or a dominant mutant DynK44A (3 μg, ■), or β-arrestin2 (600 ng, ■). (**a**,**b**) Cells were incubated in the absence or presence of 100 nM AngII for 30 min at 37 °C and then subjected to [^{125}I]-AngII binding on whole cells, as described in the Methods section. Receptor endocytosis was expressed as the percent loss of cell surface receptors. Data shown represent the means ± s.e.m. of three independent experiments. *p<0.05, **p<0.001, unpaired Student's *t*-test, compared to pcDNA transfected cells.

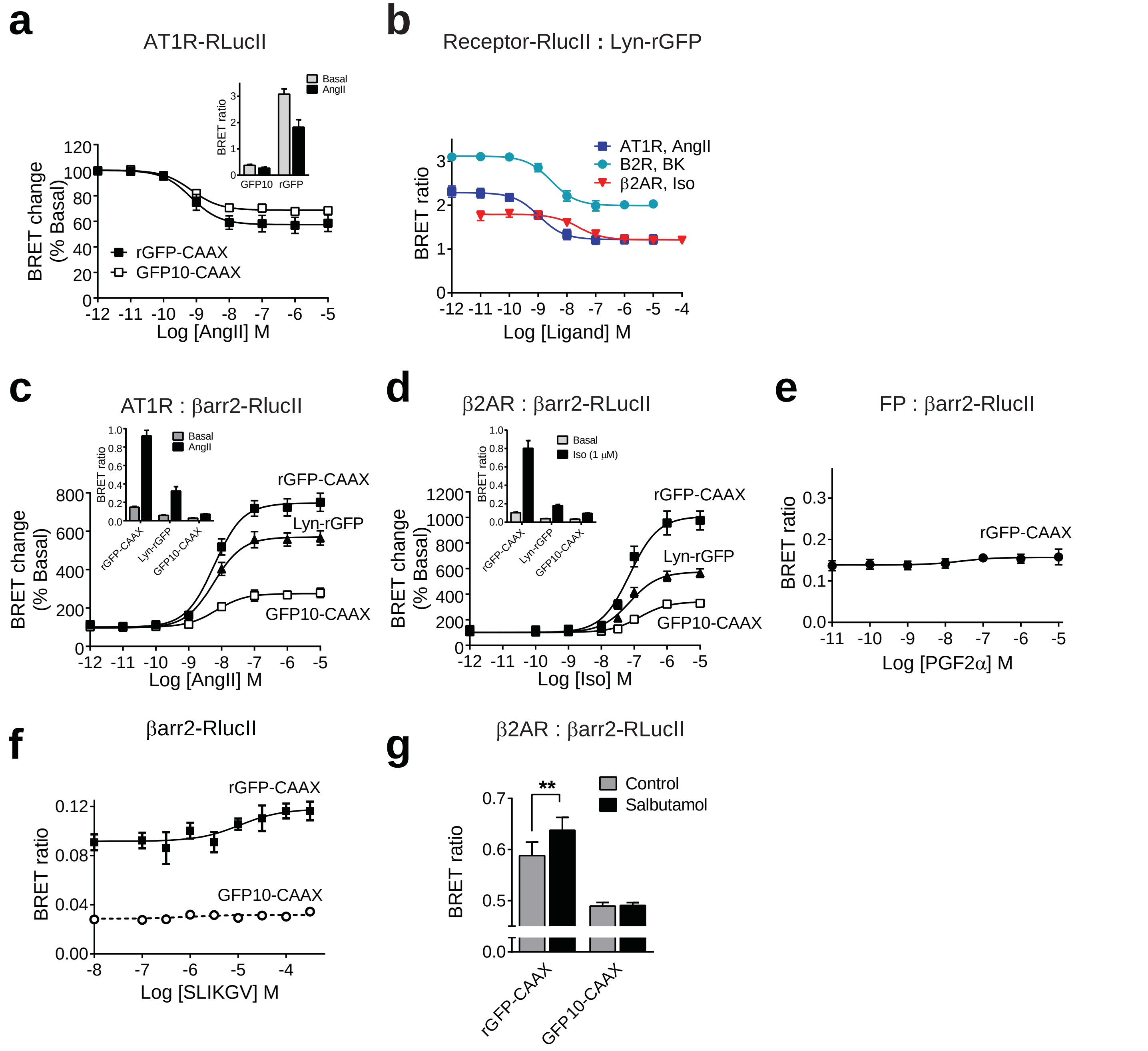

Supplementary Figure 5. Receptor and β-arrestin2 trafficking using different pairs of PM acceptor sensors, as assessed by EbBRET. (**a**,**b**) Monitoring receptor loss from the PM upon agonist stimulation using different PM-markers. (**a**) HEK293 cells co-transfected with AT1R-RLucII and either rGFP-CAAX or GFP10-CAAX were incubated with the indicated concentrations of AngII for 30 min before BRET measurements. The BRET ratio changes upon agonist treatment are calculated as in Fig. 2 and expressed as a percentage of BRET ratio observed in the control (without AngII) group. (**a, inset**) Raw BRET ratios in the absence (basal) or presence of 100 nM AngII were plotted as bar graphs. (**b**) HEK293 cells co-transfected with Lyn-rGFP and either AT1R-RlucII, B2R-RlucII, or β2AR-RlucII were stimulated with their cognate agonists (AngII, BK, and isoproterenol (Iso), respectively) at the indicated concentrations for 30 min before BRET measurements. (**c**,**d**) Monitoring of β-arrestin2 translocation to the PM upon receptor activation. HEK293 cells were transfected with βarr2-RlucII along with either rGFP-CAAX, Lyn-rGFP, or GFP10-CAAX in the presence of AT1R (**c**) or β2AR (**d**). Cells were stimulated with the indicated concentrations of ligands (AngII and isoproterenol (Iso), respectively) for 6 and 10 min, respectively, before BRET measurements. Data are expressed and presented as in a. (**e**) HEK293 cells transfected with βarr2-RlucII and rGFP-CAAX, along with the prostaglandin-F2α receptor (FP), were stimulated with the indicated concentrations of PGF2α for 10 min before BRET measurement. (**f**) HEK293 cells expressing βarr2-RlucII along with either rGFP-CAAX or GFP10-CAAX were stimulated with the indicated concentrations of SLIGKV-NH2 for 4 min before BRET measurement. (**g**) HEK293 cells were transfected with β2AR and βarr2-RlucII along with either rGFP-CAAX or GFP10-CAAX. Cells were incubated in the absence (control) or presence of 10 μM of salbutamol for 15 min before BRET measurement. **p < 0.01, paired Student's *t*-test. All data represent the means ± s.e.m. from at least 3 independent experiments.

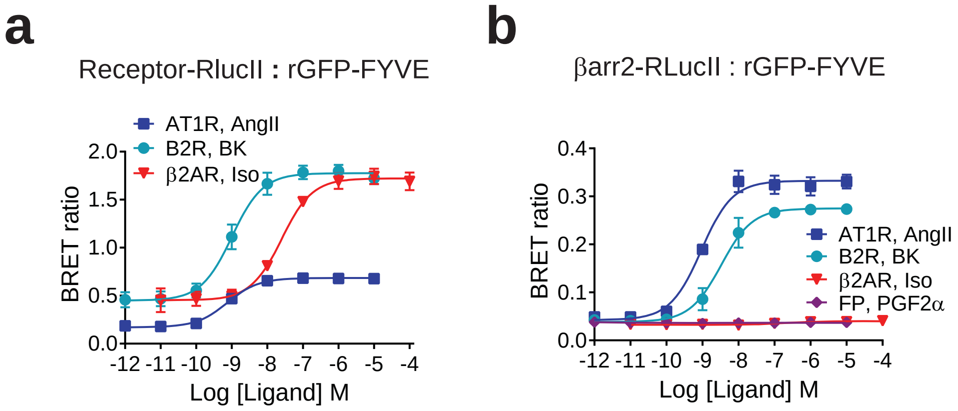

Supplementary Figure 6. Accumulation of receptors and β-arrestin2 in early endosomes (EEs) upon stimulation of different GPCRs. HEK293 cells were co-transfected with rGFP-FYVE and either (**a**) AT1R-RlucII, B2R-RlucII, or β2AR-RlucII, or (**b**) βarr2-RlucII along with 3 μg of either AT1R, B2R, β2AR, or FP. Cells were treated with the indicated concentrations of selective agonists for each receptor (angiotensin II (AngII, blue), bradykinin (BK, turquoise), isoproterenol (Iso, red), or PGF2α (purple)) for 40 min before BRET measurements. Data represent the means ± s.e.m. of raw BRET ratios obtained from 3 independent experiments.

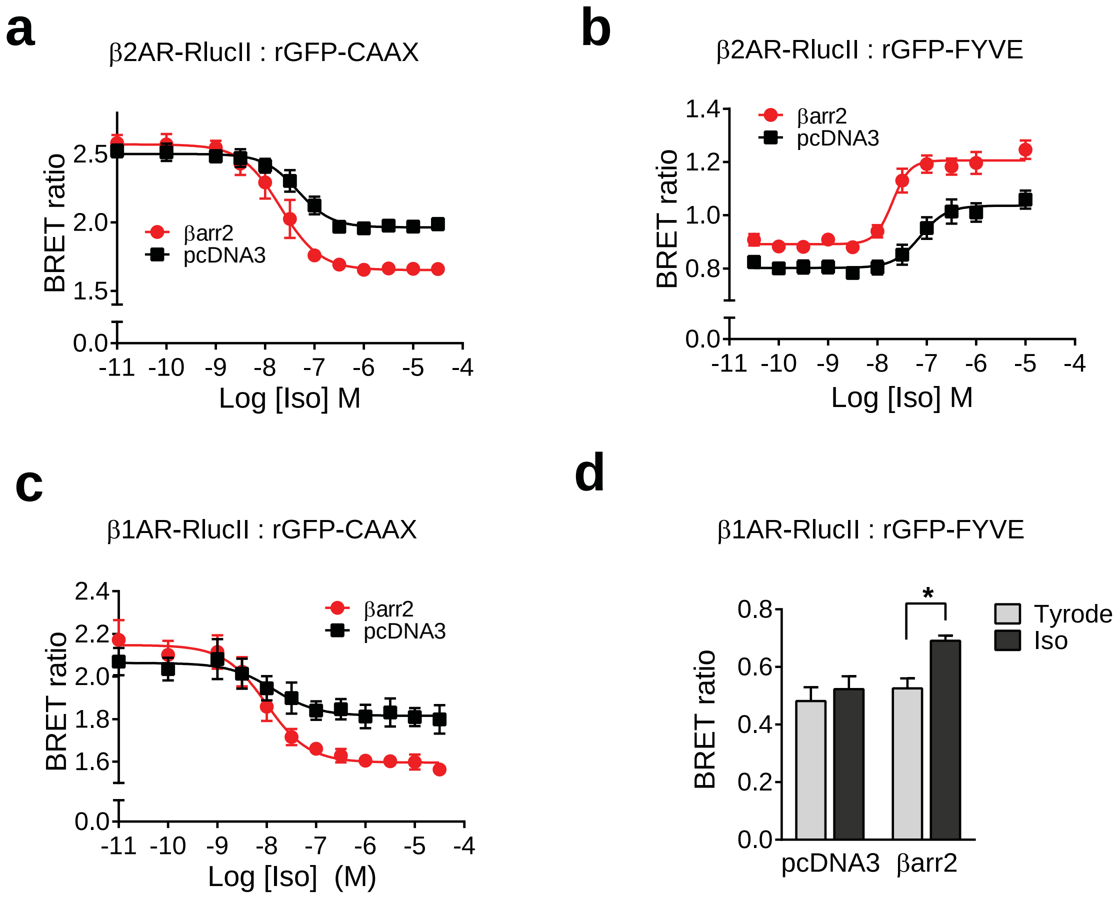

Supplementary Figure 7. Effects of β-arrestin2 on agonist-promoted endocytosis of β1AR and β2AR using PM- and EE-targeted EbBRET biosensors. HEK293 cells were transfected with rGFP-CAAX (**a**,**c**) or rGFP-FYVE (**b**,**d**) along with β2AR-RlucII (**a**,**b**) or β1AR-RlucII (**c**,**d**) in the presence (βarr2) or absence (pcDNA3) of over-expresed β-arrestin2. (**a**-**c**) Cells were treated with the indicated concentrations of isoproterenol (Iso) for 10 min before BRET measurements. For panel d, a single maximal concentration (1 μM) of isoproterenol (Iso) was used. Data represent the means ± s.e.m. of BRET ratios obtained from 3 independent experiments. *p<0.05, unpaired Student's *t*-test.

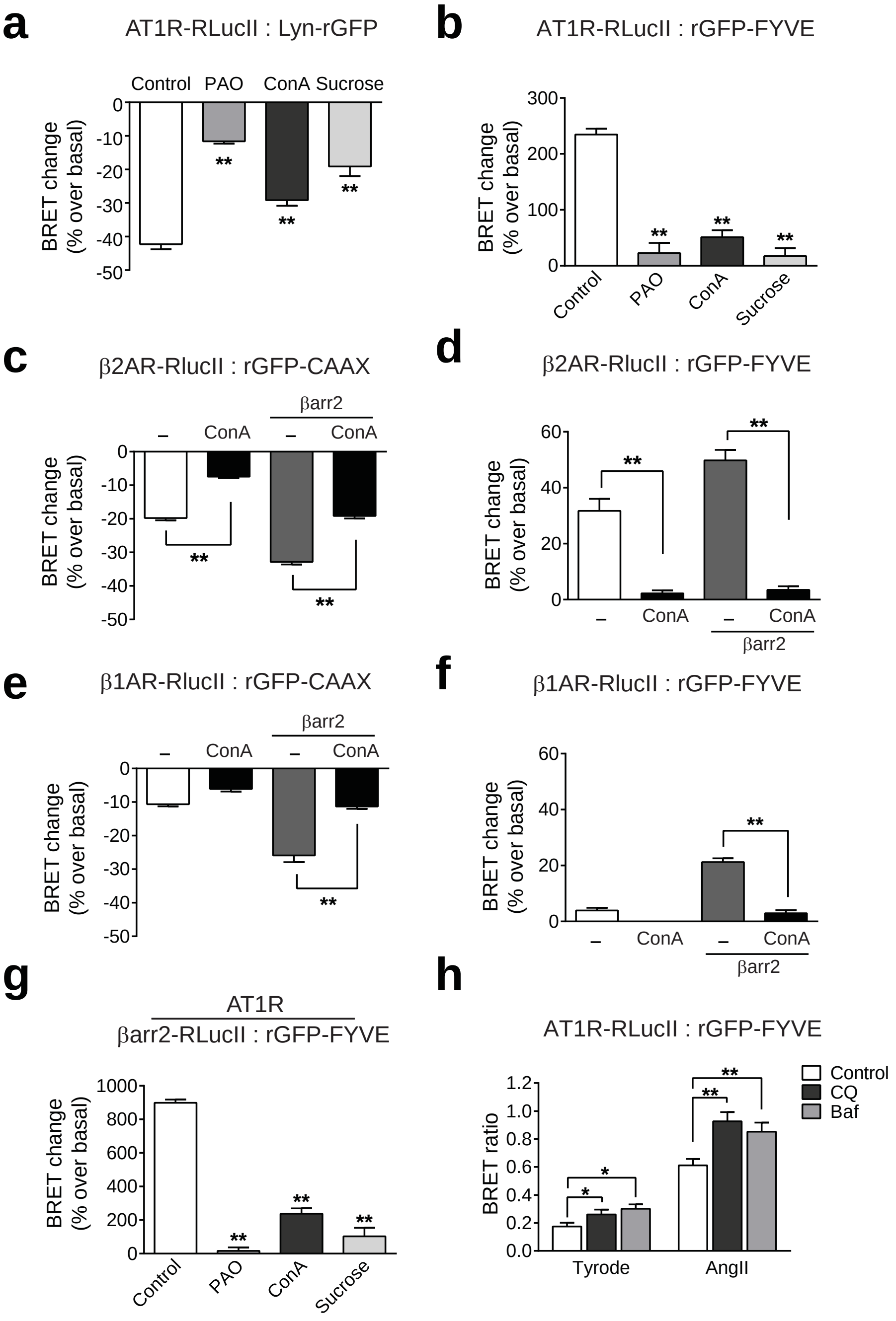

Supplementary Figure 8. Pharmacological and genetic modulation of agonist-promoted endocytosis of AT1R, β1AR, and β2AR using PM- and EE-targeted EbBRET biosensors. (**a**,**b**) HEK293 cells were transfected with AT1R-RlucII and either Lyn-rGFP (**a**) or rGFP-FYVE (**b**). Cells were incubated either in Tyrode's buffer (control), 5 μM of phenylarsine oxide (PAO), 0.25 mg ml^{-1} of concanavalin A (ConA), or 0.45 M sucrose for 30 min prior to stimulation with or without 100 nM of AngII for 30 min (**a**) or 40 min (**b**) before BRET measurement. (**c**-**f**) HEK293 cells were transfected with rGFP-CAAX (**c**,**e**) or rGFP-FYVE (**d**,**f**) along with β2AR-RlucII (**c**,**d**) or β1AR-RlucII (**e**,**f**) in the presence or absence (pcDNA) of over-expressed β-arrestin2. Cells were treated with ConA (0.1 mg ml^{-1}) or vehicle for 30 min prior to stimulation with 1 μM isoproterenol (Iso) for an additional 10 min before BRET measurements. (**g**) HEK293 cells were transfected with βarr2-RlucII, rGFP-FYVE, and AT1R. Cells were incubated either in Tyrode's buffer (control), 5 μM of PAO, 0.25 mg ml of ConA, or 0.45 M sucrose for 30 min then stimulated with or without 100 nM of AngII for 40 min. For panels **a**-**g**, agonist-promoted BRET changes are represented as percent changes from the basal BRET (no agonist) and are expressed as the means ± s.e.m. from 3 independent experiments. **p<0.001, unpaired Student's *t*-test (**a**,**b**, and **g**) or one-way ANOVA (**c**-**f**). (**h**) Cells expressing AT1R-RlucII and rGFP-FYVE were incubated either in the vehicle (DMSO, control), 50 μM chloroquine (CQ), or 200 nM bafilomycin A (Baf) for 30 min before being stimulated with 100 nM AngII or vehicle for 40 min before BRET measurements. Data represent the means ± s.e.m. of BRET ratios obtained from 3 independent experiments. *p<0.05, **p<0.001, unpaired Student's *t*-test.

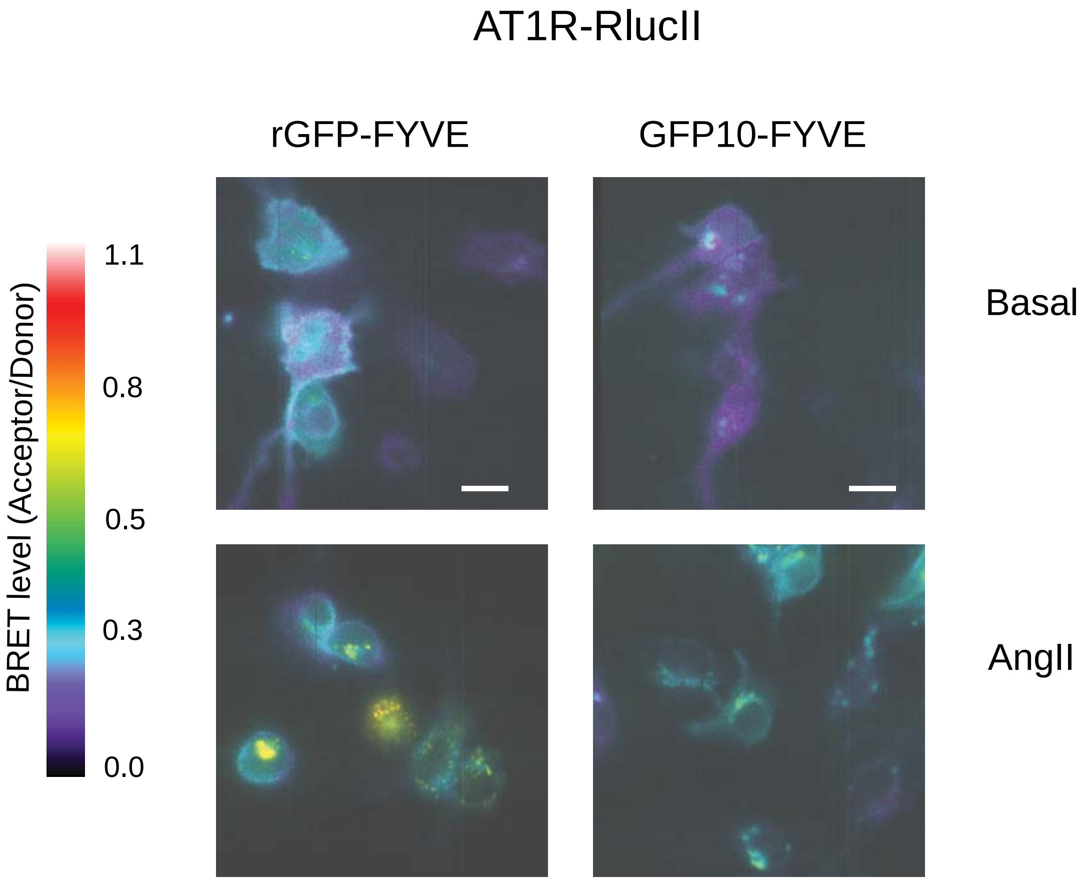

Supplementary Figure 9. EbBRET imaging of AT1R endocytosis HEK293 cells were transfected with AT1R-RlucII (100 ng) along with either rGFP-FYVE (300 ng) or GFP10-FYVE (300 ng). Luminescence images and BRET images were acquired and generated as described in Fig. 4 and the Methods section. Luciferase substrate was added to unstimulated cells (basal) or cells treated with AngII (100 nM) for 1 h prior to the acquisition of images. The numeric scale of the heat-map legend represents calculated BRET ratios. Scale bars, 20 μm.

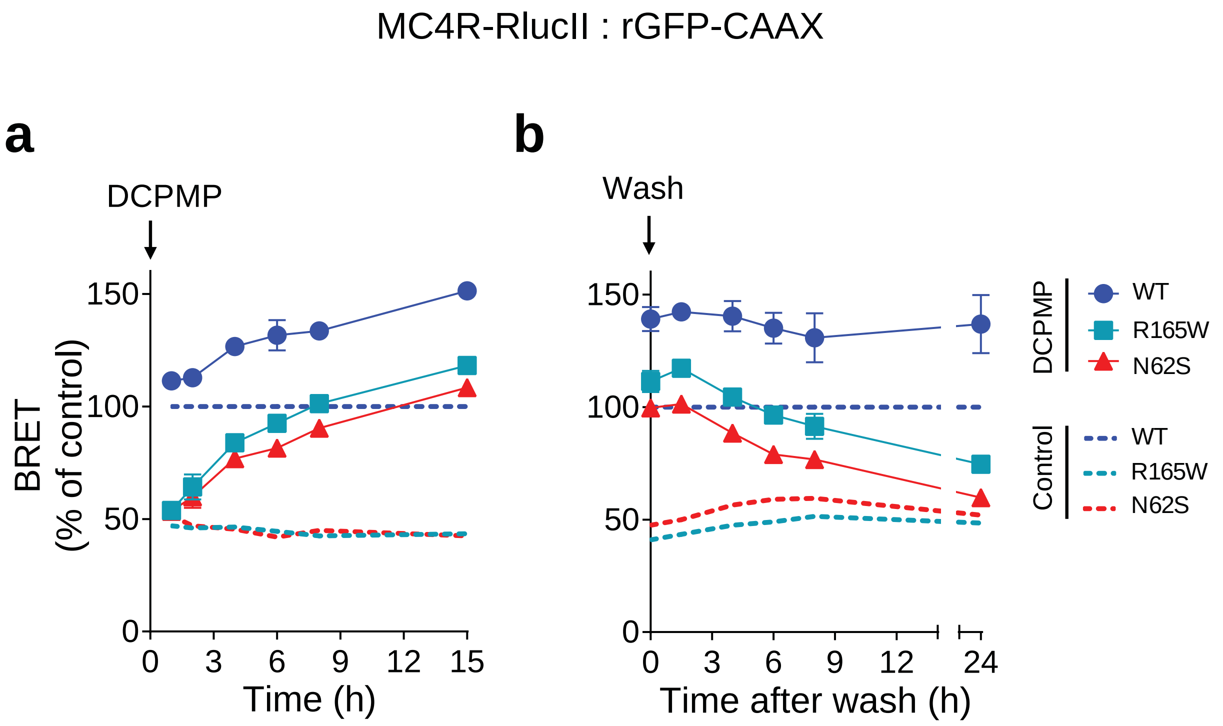

Supplementary Figure 10. Monitoring kinetics of PM expression rescue of ER-retained MC4R by a pharmacological chaperone and its reversibility. Cells were transfected with rGFP-CAAX and either WT-MC4R-RlucII (blue), R165W-MC4R-RlucII (turquoise) or N62S-MC4R-RlucII (red). (**a**) Cells were treated with 100 µM of the pharmacological chaperone (PC), DCPMP, for the indicated times before BRET measurements. (**b**) Following a treatment of 16 h with DCPMP, cells were washed extensively and incubated for the indicated periods of time in the absence of the PC. The dotted lines represent the BRET values for R165-MC4R-RlucII (turquoise) and N62S-MC4R-RlucII (red) in the absence of DCPMP treatment. BRET values are expressed as a percent of the BRET value obtained for the WT-MC4R-RlucII in the absence of DCPMP treatment (control). Data represent means ± s.d. from two independent experiments.

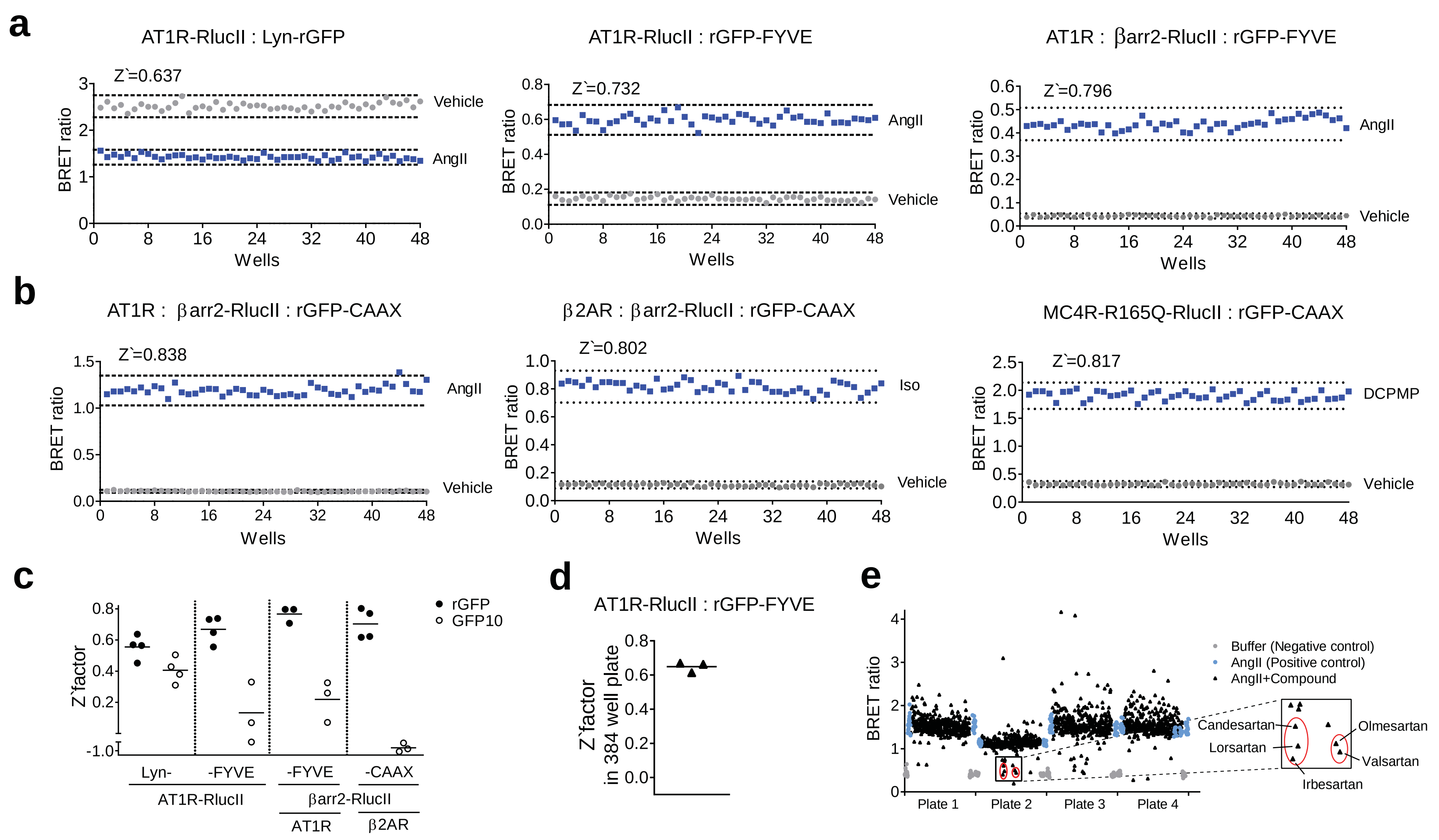

Supplementary Figure 11. Determination of the robustness and reproducibility of EbBRET. (**a,b**) Representative Z` factor data for different EbBRET assays. HEK293 cells were transfected with the indicated EbBRET biosensor pairs and plated in 96-well plates. Each well of the 96-well plate contained cells expressing the same EbBRET biosensor pair and was read individually in the presence or absence of treatment with the indicated drugs (angiotensin II (AngII, 100 nM), isoproterenol (Iso, 10 μM), and DCPMP, 100 μM). The treatment time with drugs before BRET measurements was as follows: AT1R-RlucII/Lyn-rGFP: 30 min; AT1R-RlucII/rGFP-FYVE and βarr2-Rluc/rGFP-FYVE: 40 min; βarr2-RlucII/rGFP-CAAX: 6 and 10 min when stimulated by AngII or Iso, respectively; R165W-MC4R-RlucII: 16 h. Z`-factor was calculated described in Zhang et al. Data are from each representative experiment. (**c**) Comparison of Z` factors between sensors with rGFP and GFP10 in panel **a** and **b** *middle*. Each point represent single experiment. Lines are the average Z` factors. (**d,e**) HEK293 cells were transfected with AT1R-RlucII and rGFP-FYVE and plated in 384-well plate for the screening as described in the Methods section. (**d**) Z` factors from three different 384-plates were calculated and presented. Lines are the average Z` factors. (**e**) Identification of AT1R antagonists of the sartan family as revealed by a small HTS campaign. Primary screening data of 1260 compounds with controls were plotted as raw BRET ratios. AT1R antagonists of the sartan family were indicated in the inset.

Zhang, J.H., Chung, T.D. & Oldenburg, K.R. A Simple Statistical Parameter for Use in Evaluation and Validation of High Throughput Screening Assays. *J Biomol Screen* 4, 67-73 (1999)

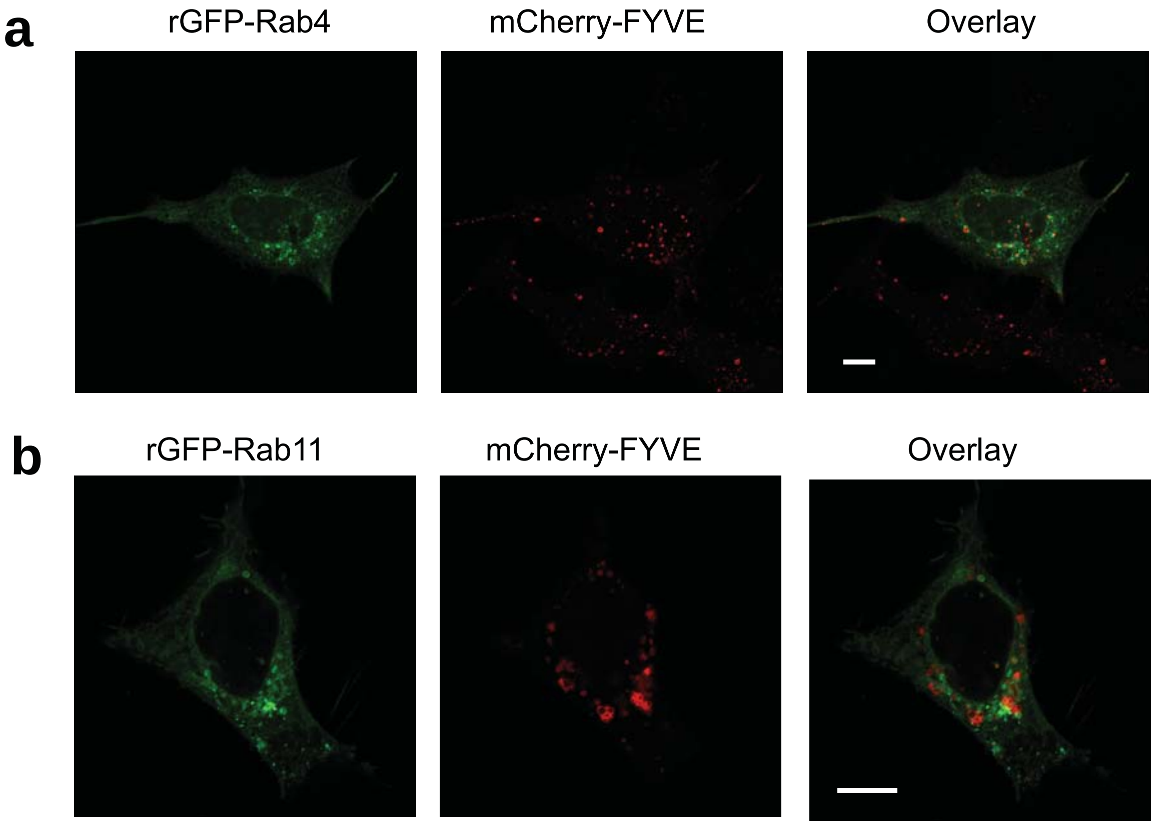

Supplementary Figure 12. Cellular localization of mCherry-FYVE, rGFP-Rab4, and rGFP-Rab11. HEK293 cells were transfected either with rGFP-Rab4 along with mCherry-FYVE (**a**) or rGFP-Rab11 along with mCherry-FYVE (**b**). Scale bars, 10 μm.
