# Supplementary material for: Monitoring G protein-coupled receptor and β-arrestin trafficking in live cells using enhanced bystander BRET
Source: Nat Commun. 2016 Jul 11;7:12178. doi: 10.1038/ncomms12178 (PMC4942582; doi:10.1038/ncomms12178)
Supplement: Supplementary Data 1 — Sequences for RlucII, rGFP, GFP10 and GFP2 [file ncomms12178-s2.docx]

**Supplementary Data 1. Nucleotide sequences of cDNAs of RlucII, rGFP, GFP10, and GFP2.**

**RlucII** ATGACCAGCAAGGTGTACGACCCCGAGCAGAGGAAGAGGATGATCACCGGCCCCCAGTGGTGGGCCAGGTGC AAGCAGATGAACGTGCTGGACAGCTTCATCAACTACTACGACAGCGAGAAGCACGCCGAGAACGCCGTGATCT TCCTGCACGGCAACGCCACTAGCAGCTACCTGTGGAGGCACGTGGTGCCCCACATCGAGCCCGTGGCCAGGTG CATCATCCCCGATCTGATCGGCATGGGCAAGAGCGGCAAGAGCGGCAACGGCAGCTACAGGCTGCTGGACCAC TACAAGTACCTGACCGCCTGGTTCGAGCTCCTGAACCTGCCCAAGAAGATCATCTTCGTGGGCCACGACTGGGG CGCCGCACTGGCCTTCCACTACAGCTACGAGCACCAGGACAAGATCAAGGCCATCGTGCACGCCGAGAGCGTG GTGGACGTGATCGAGAGCTGGGACGAGTGGCCAGACATCGAGGAGGACATCGCCCTGATCAAGAGCGAGGAG GGCGAGAAGATGGTGCTGGAGAACAACTTCTTCGTGGAGACCGTTCTGCCCAGCAAGATCATGAGAAAGCTGG AGCCCGAGGAGTTCGCCGCCTACCTGGAGCCCTTCAAGGAGAAGGGCGAGGTGAGAAGACCCACCCTGAGCT GGCCCAGAGAGATCCCCCTGGTGAAGGGCGGCAAGCCCGACGTGGTGCAGATCGTGAGAAACTACAACGCCT ACCTGAGAGCCAGCGACGACCTGCCCAAGATGTTCATCGAGAGCGACCCCGGCTTCTTCAGCAACGCCATCGT GGAGGGCGCCAAGAAGTTCCCCAACACCGAGTTCGTGAAGGTGAAGGGCCTGCACTTCAGCCAGGAGGACGC CCCCGACGAGATGGGCAAGTACATCAAGAGCTTCGTGGAGAGAGTGCTGAAGAACGAGCAGTAA

**humanized *Renilla Reniformis* GFP (rGFP)** ATGGATCTCGCAAAACTCGGTCTGAAGGAAGTCATGCCAACAAAAATCAACCTGGAAGGCCTGGTCGGCGATC ATGCATTTAGCATGGAGGGCGTCGGGGAAGGCAACATCCTGGAAGGGACACAGGAGGTAAAGATTAGTGTGAC AAAAGGAGCTCCATTGCCCTTTGCCTTTGATATCGTTAGCGTTGCGTTTAGTTATGGGAACAGGGCTTACACAGG GTACCCCGAAGAAATTTCCGACTACTTTCTGCAAAGCTTTCCTGAGGGGTTCACATACGAAAGGAATATAAGGT ATCAGGACGGGGGAACCGCGATCGTCAAATCAGATATTTCCCTCGAGGATGGTAAGTTCATCGTGAATGTGGAC TTCAAGGCGAAAGACCTGAGGCGGATGGGGCCCGTTATGCAACAGGATATTGTTGGGATGCAGCCCAGCTACG AGTCCATGTACACCAATGTGACCTCAGTGATTGGAGAATGTATCATCGCGTTTAAGCTCCAGACGGGCAAACATT TCACCTACCATATGCGCACAGTCTACAAATCCAAGAAGCCCGTCGAGACTATGCCACTGTACCACTTTATCCAAC ACCGGTTGGTGAAGACTAACGTGGATACAGCGTCAGGATACGTGGTTCAACACGAGACAGCTATCGCCGCACA CAGCACAATCAAGAAAATTGAGGGATCTTTGCCCTAG

**GFP10**

ATGGTGAGCAAGGGCGAGGAGCTGTTCACCGGGGTGGTGCCCATCCTGGTCGAGCTGGACGGCGACGTAAACG GCCACAAGTTCAGCGTGTCCGGCGAGGGCGAGGGCGATGCCACCTACGGCAAGCTGACCCTGAAGTTCATCTG CACCACCGGCAAGCTGCCCGTGCCCTGGCCCACCCTCGTGACCACCCTGAGCTACGGCGTGCAGTGCTTCAGC CGCTACCCCGACCACATGAAGCAGCACGACTTCTTCAAGTCCGCCATGCCCGAAGGCTACGTCCAGGAGCGCA CCATCTTCTTCAAGGACGACGGCAACTACAAGACCCGCGCCGAGGTGAAGTTCGAGGGCGACACCCTGGTGAA CCGCATCGAGCTGAAGGGCATCGACTTCAAGGAGGACGGCAACATCCTGGGGCACAAGCTGGAGTACAACTAC AACCCCCACAACGTCTATATCATGGCCGACAAGCAGAAGAACGGCATCAAGGTGAACTTCAAGATCCGCCACA ACATCGAGGACGGCAGCGTGCAGCTCGCCGACCACTACCAGCAGAACACCCCCATCGGCGACGGCCCCGTGCT GCTGCCCGACAACCACTACCTGTTCACCCAGTCCGCCCTGAGCAAAGACCCCAACGAGAAGCGCGATCACATG GTCCTGCTGGAGTTCGTGACCGCCGCCGGGATCACTCTCGGCATGGACGAGCTGTACAAGTAA

**GFP2**

ATGGTGAGCAAGGGCGAGGAGCTGTTCACCGGGGTGGTGCCCATCCTGGTCGAGCTGGACGGCGACGTAAACG GCCACAAGTTCAGCGTGTCCGGCGAGGGCGAGGGCGATGCCACCTACGGCAAGCTGACCCTGAAGTTCATCTG CACCACCGGCAAGCTGCCCGTGCCCTGGCCCACCCTCGTGACCACCCTGAGCTACGGCGTGCAGTGCTTCAGC CGCTACCCCGACCACATGAAGCAGCACGACTTCTTCAAGTCCGCCATGCCCGAAGGCTACGTCCAGGAGCGCA CCATCTTCTTCAAGGACGACGGCAACTACAAGACCCGCGCCGAGGTGAAGTTCGAGGGCGACACCCTGGTGAA CCGCATCGAGCTGAAGGGCATCGACTTCAAGGAGGACGGCAACATCCTGGGGCACAAGCTGGAGTACAACTAC AACAGCCACAACGTCTATATCATGGCCGACAAGCAGAAGAACGGCATCAAGGTGAACTTCAAGATCCGCCACA ACATCGAGGACGGCAGCGTGCAGCTCGCCGACCACTACCAGCAGAACACCCCCATCGGCGACGGCCCCGTGCT GCTGCCCGACAACCACTACCTGAGCACCCAGTCCGCCCTGAGCAAAGACCCCAACGAGAAGCGCGATCACATG GTCCTGCTGGAGTTCGTGACCGCCGCCGGGATCACTCTCGGCATGGACGAGCTGTACAAGTAA
